# Understanding the impact of perceived emotional valence of eco-labels for novel food products on consumer preferences, visual attention, and cognition – a multimodal study using EEG and eye tracking

**DOI:** 10.3389/fpsyg.2026.1836306

**Published:** 2026-09-08

**Authors:** Uma Parasuram, Chengyan Yue, Garrett Steede, R. Karina Gallardo, Benjamin R. Eisenreich, Matthew Clark

**Affiliations:** 1 Department of Applied Economics, University of Minnesota, Twin Cities, St. Paul, MN, United States; 2 Department of Horticultural Science, University of Minnesota, Twin Cities, St. Paul, MN, United States; 3 Agricultural Education, Communication and Marketing, University of Minnesota, Twin Cities, St. Paul, MN, United States; 4 School of Economic Sciences, Washington State University, Pullman, WA, United States; 5 Department of Neuroscience, Center for Magnetic Resonance Research, and Center for Neuroengineering, University of Minnesota, Twin Cities, St. Paul, MN, United States

**Keywords:** eco-labels, EEG, eye-tracking, sustainability, valence, wine

## Abstract

**Background:**

Eco-labels are becoming an important marketing tool used to communicate these environmental attributes to consumers. However, little is known about how the perceived emotional valence of sustainability messaging influences the cognitive process underlying the purchase decision.

**Methods:**

This exploratory study investigates the effect of perceived emotional valence of eco-labels on consumer preferences, visual attention and neural processing for novel food products by integrating behavioral (choice data), eye-tracking and EEG data..

**Results:**

Behavioral results indicated that eco-labels with high perceived positivity significantly increased purchase intentions, whereas labels with low perceived positivity did not. Eye tracking results revealed shorter fixation durations and lower number of fixations for high perceived positivity eco-labels, possibly suggesting greater processing fluency. The EEG results further revealed differences in P300 and LPP amplitudes for the different label categories. Integrated behavioral-neural analyses further showed that higher P300 amplitudes increased purchase intentions, whereas greater N200 amplitudes reduced purchase intentions for high-positivity labels.

**Conclusion:**

The results provide preliminary evidence showing that the effectiveness of eco-labels depends not only on what is communicated but how it is perceived. By conceptualizing valence as a continuum rather than a binary positive–negative construct, this study offers new hypotheses regarding the attentional and neural mechanisms through which sustainability communication shapes consumer preferences.

## Introduction

Sustainability has become an important focal point in the food and beverage market today; particularly eco-labels have become an important marketing tool to communicate environmental attributes to consumers. Labels like “organic” and “fair trade” help reduce information asymmetries, increase trust and promote environmentally friendly purchase behavior (Testa et al., 2015). A growing body of research has shown that consumers are often willing to pay more for products with eco-labels (Duckworth et al., 2022; Loureiro and Hine, 2002; Van Loo et al., 2014). However, the effectiveness of these labels depends on the how the message is framed, familiarity and alignment of the messages with consumer’s expectations (Fischer et al., 2021; Ropret Homar and Knežević Cvelbar, 2021).

The present study proposes perceived emotional valence as a central mechanism through which sustainability messages influence consumer responses. Although valence framing or more generally message framing of environmental messages has been an effective strategy to increase the salience of such messages (Cucchiara et al., 2015; Grazzini et al., 2018; Ropret Homar and Knežević Cvelbar, 2021), prior research has focused primarily on how messages are objectively framed as gains or losses, positive or negative, or self- versus other-oriented (Ropret Homar and Knežević Cvelbar, 2021). Emotional valence, on the other hand, is the feeling of pleasant or unpleasant (or negative and positive) emotion towards a stimulus and is commonly represented as a continuum ranging from negative to positive affect (Russell, 1980). Much of the environmental-framing literature has examined objectively constructed gain versus loss or positive versus negative messages, drawing on prospect theory and loss aversion (Kahneman and Tversky, 1979). These studies show that alternative presentations of substantively similar information can affect environmental attitudes and behavior, although the relative effectiveness of positive and negative frames remains mixed across contexts (Ropret Homar and Knežević Cvelbar, 2021).

Most of the existing research on valence framing is based on self-reported measures of preference and attitudes, which provide limited insights into the underlying cognitive and emotional mechanism. Further, the mixed results of message framing could be attributed to the binary treatment of valence, and recent research has called for a more fine-grained approach to studying valence framing. A nominally positive environmental label may be viewed as unfamiliar, technical, or ambiguous and may therefore evoke less positive effect than expected. Carfora et al. (2022), for example, showed that consumers’ subjective evaluations of message valence helped explain responses to local-food framing. Similarly, Dorisse et al. (2025) found that the perceived coherence and valence of multiple sustainability labels affected confusion and purchase intentions. These findings suggest that the perceived emotional valence, rather than the strict binary framing alone, may be important for understanding the effectiveness of sustainability communication.

The challenge is particularly salient when communicating novel sustainability attributes of emerging technologies. There is a growing effort within the wine industry to reduce its reliance on chemical pesticides and fungicides by growing grape varieties that are naturally resistant to common fungal diseases like downy mildew and powdery mildew (Borrello et al., 2021). Cross breeding these grape species and varieties with commercial varieties of grapes gives rise to ‘disease-resistant varieties’ (DRV) of grapes. Wines made from such grapes offer multiple benefits such as reduced dependence on synthetic pesticides by up to 80% (Borrello et al., 2024), improved soil health and biodiversity, and improved conditions for the grape breeders (Pedneault and Provost, 2016). However, there is very low consumer awareness regarding DRV wines and terms like ‘disease resistant’ may increase consumer skepticism which may lead to the sustainability liability effect (Turut, 2024). Furthermore, wines are typically identified by grape variety rather than production method. As DRV wines become more prevalent, consumers may find it increasingly difficult to distinguish them from conventional wines based solely on varietal names (Borrello et al., 2024). This suggests a potential role for a unified eco-label that readily signals that the wine is a DRV to consumers at the point of sales. As stated by Tait et al. (2019), a key challenge of the wine sector and agri-food industry is developing communication strategies that make sustainability attributes credible and understandable. Thus, developing communication strategies may be of great value to companies and the wine industry generally.

Although the behavioral effects of eco-labels have been widely documented, relatively little is known about the cognitive mechanisms through which different sustainability messages influence consumer decisions. Recent literature reviews on consumer neuroscience and behavioral science have highlighted the rapid growth of neuroscience methods like electroencephalography (EEG) and eye-tracking (Ariely and Berns, 2010; Duerrschmid and Danner, 2018) to study sustainable consumption, emphasizing the need for studies that integrate multiple physiological measures to better explain the mechanisms underlying sustainable decision-making (Liu et al., 2023; Richelli et al., 2025). Existing neuroscience studies have primarily compared eco-labeled and non-labeled products, examined positive versus negative framing, or investigated the effects of packaging and visual design on consumer responses (Seo et al., 2025; Wang et al., 2021; Zubair et al., 2020). While these studies demonstrate that sustainability cues can reduce cognitive conflict and influence preferences, they provide limited insight into why some sustainability messages are more persuasive than others when all communicate environmentally beneficial attributes. Behavioral measures alone reveal what consumers choose but provide limited insight into how those choices are formed. By combining behavioral responses with eye-tracking and EEG, it becomes possible to examine complementary stages of decision-making, including visual attention, conflict monitoring, and evaluative processing.

Accordingly, this exploratory study addresses the following research questions: How is the perceived emotional valence of eco-labels associated with consumers’ purchase intentions for wines made from disease-resistant grape varieties? Do eco-labels with different levels of perceived emotional valence differ in the visual attention they receive? Do eco-labels with different levels of perceived positivity elicit differences in ERP components associated with early visual processing, cognitive conflict, evaluative attention, and sustained emotional engagement? And how are visual-attention and neural-processing measures associated with purchase intentions across eco-labels with different levels of perceived positivity?

By integrating behavioral, attentional, and neural measures, this research contributes to three streams of literature. First, it advances valence framing research by measuring valence as a continuum between very negative to very positive rather than a simple binary positive and negative dichotomy. Second, this study demonstrates the value of conducting multi-modal research that connects preference formation with its underlying cognitive and affective mechanisms. Third, it extends the literature on consumer preferences and marketing strategies for DRV wines.

Given the novelty of applying a multimodal approach to study perceived emotional valence of sustainability labels, this research should be viewed as an exploratory investigation of the cognitive and attentional processes underlying consumer responses. Rather than providing definitive evidence of these mechanisms, the integration of behavioral, eye-tracking, and EEG measures is intended to generate and refine hypotheses regarding how perceived valence influences information processing and purchase intentions. These hypotheses provide a foundation for future confirmatory studies using larger and more diverse samples.

## Literature review

### Food eco-labeling

There is increasing literature on the effectiveness of front-of-package (FoP) food labels to influence purchase and consumption behavior (Tiboni-Oschilewski et al., 2024). Labels are claims provided by the seller describing characteristics of the product that can help reduce information asymmetries between producers and consumers (de Boer, 2003). Specifically, brands and governmental organizations have increased the use of eco-labeling as an effective market instrument to increase the consumption of environmentally friendly products (Hou et al., 2023).

Eco-labels serve as effective visual cues to communicate environmental attributes of a product, supporting the sustainability evaluation of the product prior to purchase (Dorisse et al., 2025; Testa et al., 2015; Tiboni-Oschilewski et al., 2024). They also reduce uncertainty by providing understandable information and have also shown to promote change in consumer purchasing behavior (Thøgersen and Nielsen, 2016). For example, Duckworth et al. (2022) explored UK consumer purchasing decisions for food products with green labels. They found that when compared to no label, consumers preferred products with green labels. Furthermore, when the labels were directly compared with each other, consumers significantly preferred and were willing to pay more for the ‘sustainably sourced’ and ‘locally sourced’ labels. Similarly, Thøgersen and Nielsen (2016) studied the effectiveness of carbon footprint labels on consumer’s preferences for coffee and found that these labels had a significant impact on consumer’s choices. Extending these labels with a simple three-tier traffic light ranking amplified its effectiveness, shifting consumer choices more towards low carbon intensive coffee products.

More specifically, eco-labeling within the wine industry has also received growing attention with the increased focus on sustainability and changing consumer preferences. A recent systematic review by Anagnostou et al. (2025) concluded that while consumers are interested in sustainability certifications, there is limited understanding of these certifications among consumers which can decrease their impact on purchase decisions. Some studies have also shown that consumers viewed organic wines as premium and were willing to pay more but some others link sustainability attributes like organic to poorer quality (Delmas and Gergaud, 2021). However, many studies have highlighted the positive impacts of eco-labels on wine preferences (Borrello et al., 2024; Sogari et al., 2016) and labels emphasizing sustainability and biodiversity can strengthen consumer trust (Mazzocchi et al., 2019). The effectiveness and acceptance of the same depends on consumer’s ability to understand them, how well they align with consumer’s values and the transparency of eco-labels (Rahman et al., 2014; Ugaglia et al., 2021). Fantechi et al. (2025) used a discrete choice experiment to evaluate Italian consumer’s preferences for four eco labeled wines including organic, carbon neutral, reduced water footprint and reduced pesticide use. They found heterogeneous preferences and identified four distinct classes of consumers; some were indifferent towards eco-labeled wines while two segments showed strong preferences depending on the environmental dimension highlighted by the label. They found that reduced pesticides and reduced water footprint labels were valued due to its personal and health related relevance to people. Overall, prior research has shown that eco-labels can decrease information asymmetries and increase consumer trust. The effectiveness varies across consumers, perceived relevance, clarity and alignment with personal values. These findings highlight the need to better understand how various sustainability related cues are processed.

### Valence, framing and consumer behavior

Previous literature on eco-labels have highlighted the importance of communication on the perception of the labels and consumer preferences. Communication strategy is pivotal especially for novel sustainability claims, as unlike institutionalized labels (like USDA Organic), unfamiliarity could trigger a “sustainability liability” bias, increasing skepticism and ambiguity (Luchs et al., 2010). Within this context, past research has studied the impact of emotional valence broadly and valence framing particularly as “nudges” to increase the salience of sustainability messaging to improve pro-social behavior. This stems from prospect theory that has shown that people are more sensitive to losses than equivalent gains, a phenomenon known as loss aversion (Kahneman and Tversky, 1979; Tversky and Kahneman, 1992). Therefore, objectively equivalent messaging can elicit different responses based on the presentation of the information (Thaler et al., 1997).

This framing effect has been widely used to improve health messaging, increase pro-environmental behaviors and improve the marketing communications of sustainable and local foods. However, the application of valence framing to environmental messages while growing, the results remain mixed. For instance, Cucchiara et al. (2015) studied the effects of positively and negatively framed messages on consumer’s purchase intentions for organic seafood in the US and found that positive messages outperformed negative messages in persuading consumers to buy organic food. On the other hand, Grazzini et al. (2018) studied the effects of gain and loss framing of messages to increase recycling among hotel guests using a field and laboratory experiment. They found that guests were more likely to engage in recycling behavior when the messages were paired with a loss framed message. Ropret Homar and Knežević Cvelbar (2021) conducted a systematic review of the literature on the effects of framing on environmental decisions and found mixed results in the literature which is often based on stated preference studies. They found that gain frames are effective in changing environmental beliefs and attitudes while loss framing to be more effective in changing behavior. These differences could come from several factors some of which may be the product type, purchase involvement, environmental attitudes and credibility of the messages.

A growing line of research has shown that the effectiveness of framing increases when it matches the orientation of the product. Weisstein et al. (2024) found that willingness to pay for sustainable foods increased when hedonic products were paired with self-focused claims (like health and taste) and utilitarian products were paired with environment focused claims. These effects were mediated by perceived taste and quality. Similarly, Lee and Cho (2022) found that gain framed messages along with positive valence images increased advertising and brand attitudes compared to loss framing with negative valence images.

Studies have noted that the mixed results on the effects of valence framing can be attributed to, in some part, on the treatment of valence as binary (positive vs. negative), which may not be fine- grained enough (Carfora et al., 2022). Carfora et al. (2022) used a more fine-grained classification of valence framing by presenting messages related to local food in four frames – gain, non-gain, loss and non-loss and measured message tone on a negative–positive scale. They found that subjective valence perceptions mediated the effects of framing. Particularly, pro-environmental consumers responded better to information regarding the environmental consequences of buying local food in the gain/ non-gain frame. While health-driven consumers responded more to the loss/non-loss frame.

Going beyond message framing using textual messages, Dorisse et al. (2025) studied the effect of valence framing and orientation of multiple eco-labels on the front of the package for food products. They found that labels with conflicting valence or orientation increased confusion and decreased purchase intention, compared to a label with a single positive valence eco-label. The results of this study highlighted that labels are usually easy to read and generally trigger System 1 thinking (fast and automatic processing), but conflicting and inconsistent labels require more effortful System 2 (more deliberate, slower and effortful) processing (Kahneman, 2011).

### Eye tracking, visual attention, and valence

Traditional research in consumer behavior has relied largely on self-reported methods such as interviews, surveys, etc. which may fail to capture implicit or more automatic processes related to the purchase decision (Alvino et al., 2021; Ariely and Berns, 2010). Moreover, consumer preferences are often shaped by subconscious attentional and emotional processes which may be difficult to articulate. Neuroscience tools like eye-tracking and EEG have been useful to study these automatic processes more closely (Bojko, 2013; Duerrschmid and Danner, 2018). Eye tracking uses sensors and cameras to track where the consumer is looking and for how long while viewing different stimuli, and is a useful method to measure people’s visual attention. Information that has been attended to can influence choice (Orquin and Mueller Loose, 2013) and eye-tracking measures like fixations, glances, heat maps and scan paths that capture visual attention have been related to preferences and choice (Reutskaja et al., 2011).

A growing body of work has studied the effectiveness of sustainability labels using eye tracking. In a systematic review by Hoffmann et al. (2025) on eye-tracking studies for sustainable labels, shape, color, position and representation of labels were found to affect visual attention which was related to their final choice (Van Loo et al., 2015). Specifically, pictorial or logo-based cues attract more attention than text-based claims (Fernández-Serrano et al., 2022; Katz et al., 2019) and claims that are new or unfamiliar like “hormone-free” received longer gaze durations compared to more familiar attributes like price and brand (Grebitus et al., 2015).

In the wine context, Fernández-Serrano et al. (2022) found that consumers highly preferred “sustainable irrigation (SI)” labeled wines which captured more attention. Particularly, they found that the fixation counts, and total duration of fixations were higher for the labels in logo form compared to text form on the front label. Similarly, Liu et al. (2022) studied the preference of representing the country of origin information on the wine label in New Zealand, who found that logos elicited higher fixations and a stronger emotional reaction compared to textual claims. Extending this, Van Loo et al. (2015) measured attention towards sustainable labels on coffee by measuring fixation time and count and found that attention significantly predicted consumer’s stated importance of the label and their willingness to pay. In a subsequent study on preference for nutrition and sustainability claims on granola bars using a choice experiment, Van Loo et al. (2021) found that nutritional information, non-genetically modified (non-GMO) logo and sustainability claims were attended the most. Further, higher visual attention (measured as visit duration) was associated with a higher choice likelihood.

Recent literature has also studied the influence of emotional tone or valence of stimuli on visual attention. In an online study, Chen et al. (2022) found that negative reviews on a product elicited longer fixation durations and number of fixations compared to positive reviews. The finding is consistent with previous literature on the negativity bias in cognition (Rozin and Royzman, 2001; Soroka et al., 2019) that states that information that is ambiguous or negative often incur deeper evaluation and consideration. Similarly, Kohout et al. (2023) observed that while reading social media comments, negatively valenced comments capture more attention. Usée et al. (2020) studied the effect of positive and negative valence vignettes and pictures using eye tracking and found that texts with perceived positive valence had shorter reading times than perceived negative valenced texts.

Together, these findings indicate that eco-labels attract higher visual attention, and the valence of stimuli also modulates cognitive effort. Building on this evidence we expect the eco-labels in this study to incur more attention compared to other label attributes. Further, the disease-resistant eco-labels with higher perceived positive valence or familiarity could elicit shorter fixation durations and number of fixations compared to less familiar badges, reflecting greater processing fluency.

### Neural correlates of valence and cognitive processing (EEG evidence)

Recent research has used electroencephalography (EEG) to study the effects of eco-labeling and message framing on cognitive processing and preferences. Event-related potentials (ERPs) are often used as the EEG metrics to measure such effects. ERPs are “a set of voltage changes that are consistent with a single generator site and that systematically vary in amplitude across conditions, time, individuals, and so forth and a source of systematic and reliable variability in an ERP data set” (Luck, 2014). ERP components differ in their timing and functional meaning; early components usually occur after the stimulus onset until about 300 milliseconds (ms) represent automatic responses while components after 300 ms like the N400 and late positive potential (LPP) represent more conscious processing (Ozkara and Bagozzi, 2021). Previous research has used these techniques to study how message framing and valence influence cognitive and affective responses to sustainability-related messages and product cues (Wei et al., 2024; Zubair et al., 2020). Some of these studies focused on consumer preferences for eco-labeling and messaging. Jin et al. (2018) reported smaller P200 and N200 for eco-labeled food compared to non-labeled food, which was interpreted as eco-labels having a more positive effect and reduced cognitive conflict. Zubair et al. (2020) studied the effects of positive and negative framing in the context of green marketing and found that participants preferred positively framed messaged. They found that negative messages elicited higher P100 amplitudes, meaning they attracted more attentional resources, while the N170 and P300 components were higher for positively framed messages, indicated increased motivational salience towards these messages. Similarly, Wei et al. (2024) studied the effects of message framing on utilitarian and hedonic products using ERPs. They found that for hedonic products, the P200 and LPP amplitudes were significantly higher for negative frames compared to positive frames, which indicated that negative frames evoked deeper emotional uncertainty and risk. Recent research has also begun to explore valence as a continuum rather than a binary positive and negative contrast. For example, Onishi and Nakagawa (2019) varied auditory stimuli from very negative to very positive and found that both extreme valences (very negative and very positive) elicited stronger P300 responses compared to neutral toned, indicating that emotionally charged stimuli command greater cognitive resources.

Thus, there is evidence that eco-labels and the valence of labels can affect both automatic and more conscious processing. We extend prior research by investigating the impact of perceived valence as a continuum on the neural processing for visual stimuli by evaluating the differences in various ERP components, particularly eco-labels for novel food products.

## Methods

### Sample

Two main criteria for eligibility in the study were that participants had to be at least 21 years or older and consumed wine at least once in the past year. All participants had normal or corrected-to-normal vision, they reported no history of neurological or major medical or psychiatric disorder or hearing impairments in their past. Participants were asked if they were comfortable sitting and focusing on a task for an hour. As the study involved alcohol-related stimuli, we used three questions from the alcohol use disorders test (AUDIT) to exclude participants at the risk of alcohol dependence and only individuals in the low-risk zone (those who scored between 0 and 4) were eligible. A total of 18 participants were recruited through multiple channels such as advertisements on social media and word of mouth. The total time of the experiment, including equipment set up and the post experiment survey was approximately 1 h and 20 min. Data was collected during the first 2 weeks of December 2024 in St. Paul, MN. A quota-based sampling strategy was used to ensure the participants represent the demographics of US wine consumers on variables like age, gender, income and education. Each participant received $60 for their time. After cleaning the data and accounting for incomplete responses, we were left with 13 participants which made up the sample for this study.

### Label and experiment design

#### Label design

To create the front of the bottle wine labels (stimuli) we took a multistage approach, which involved qualitative focus groups, a large-scale qualitative survey, consultation with industry experts, and literature reviews on wine labels. First, a large-scale open-ended survey was conducted across wine consumers in the US (*N* = 500) during November and December 2023 to understand consumer sentiment regarding wines made from disease-resistant grapes. The survey results helped identify commonly used and preferred phrases to describe disease-resistant grapes. Using these results, the research team identified top phrases that communicated various aspects of disease resistance and then consulted with industry stakeholders and scientists in the field to refine these. This process resulted in the five phrases that were then used as badges and two creative phrases. The results from the qualitative survey also revealed that while consumers were generally optimistic about using disease-resistant grapes, they expressed some concerns about these grapes being genetically modified. These concerns are similar to results from Borrello et al. (2021) who found lower willingness to pay (WTP) among consumers who associated fungus-resistant varieties of grapes with GMOs. We therefore included the non-GMO logo to the wine label.

Research has shown that consumers prefer sustainability attributes presented as images rather than plain text and images also result in increased visual attention (Fernández-Serrano et al., 2022; Hoffmann et al., 2025). We therefore designed five badges (eco-labels) using the finalized phrases to add to the wine labels to communicate disease resistance to consumers. The other label attributes such as volume, alcohol percentage, grape variety, were based on a literature review on the important attributes consumers considered while evaluating the front of a wine label (Lockshin and Corsi, 2012) and laws regarding what needs to be shown on a wine label stated by the Alcohol and Tobacco Tax and Trade Bureau.

**Figure 1:**
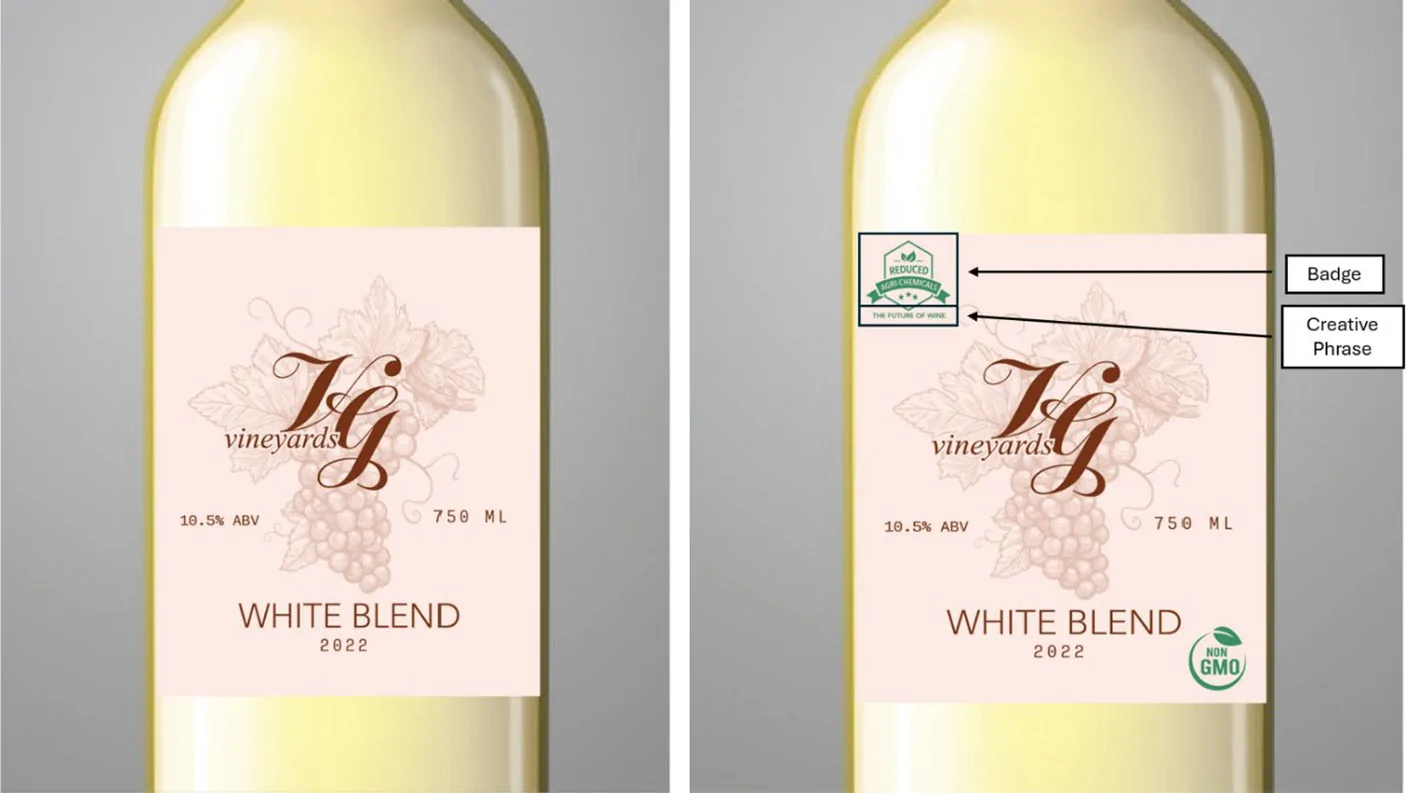
Sample label designs used in the experiment. The base condition (left) and one of the labels with disease-resistant attributes, creative phrase and the non-GMO logo (right).

**Figure 2:**
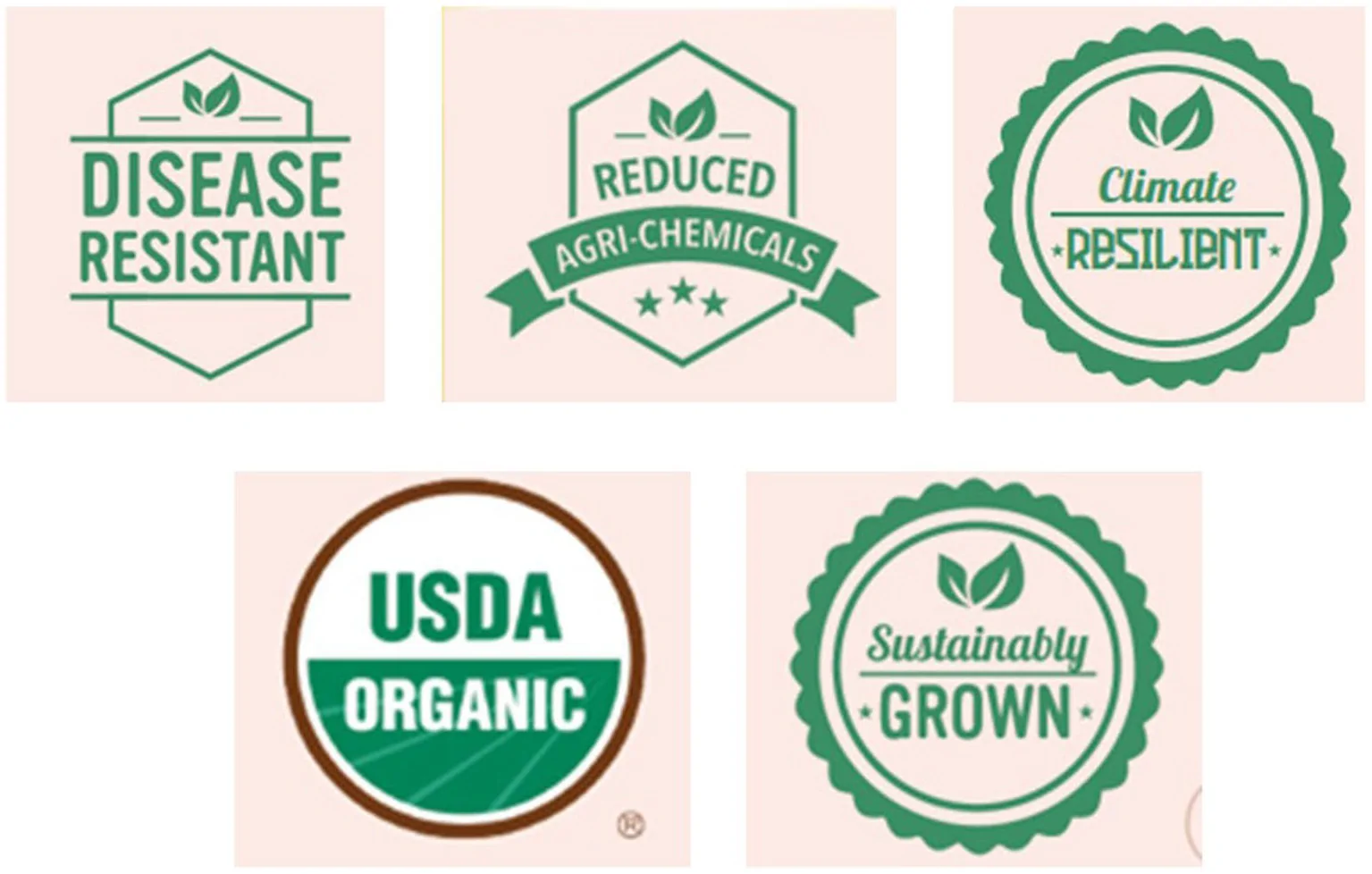
The five eco-labels (badges) used on the wine label.

A fictitious brand called VG Vineyards was created for the purpose of this study as we did not want the choices to be influenced by brand since this was not the purpose of the study. To control label design influence, all wine labels were made to look visually similar and badges and creative phrases were attributes that varied. A neutral green color scheme was chosen to minimize color-based biases and align with eco-friendly themes and the USDA organic label. While the positioning of the various label attributes was beyond the scope of this research and we kept the positioning of the various label attributes constant, which was based on previous eye-tracking research. Research has shown that consumers often process stimuli which has minimal text and a balanced design in a Z-pattern, starting from the top left corner moving across the top right and then diagonally to the bottom left (Babich, 2017; Buscher et al., 2009). Further, research on organic labels has shown that placing it on the shoulder and top of the bottles captured gaze first (Ozturk et al., 2023). Figures 1, 2 depict the label design and the five badges used in this study, respectively.

#### Post-hoc survey

In order to understand how consumers perceived the emotional valence of the different badges communicating disease resistance, we conducted a post-hoc survey that included questions regarding the perceived valence of the badges with an independent sample of 27 wine consumers. The survey was designed after the main EEG and eye-tracking experiment based on insights from a focus-group study to understand consumer’s perceptions towards wines made from disease-resistant grapes (Steede et al., 2026). The results from the focus group revealed that consumers differed in the way they interpreted sustainability related messaging on wine labels. Particularly, the focus group participants suggested the use of familiar and positively valanced terms to describe disease resistance using terms like “sustainable,” “chemical-free,” or “environmentally friendly.” They were also more skeptical towards technical and scientific terms like “disease resistant” which were viewed as clinical and negative. These findings highlighted that the emotional tone or valence of the label may shape consumer’s perceptions towards novel sustainability attributes even if they all communicated different aspects of disease resistance. We therefore asked participants to rate the five badges on a 7-point likert scale (1 = very negative, 7 = very positive) to capture perceived emotional valence. This question is similar to the one used by Carfora et al. (2022) to measure the valence of the messages used in their experiment.

#### Experiment design

A full factorial experiment design was employed to design the various labels using three attributes: Badge type (6 levels), creative phrase (3 levels) and non-GMO logo (2 levels) (Table 1). A full factorial design (6 × 3 × 2) would result in 36 unique combinations. To ensure logical coherence and relevance (e.g., not to include a creative phrase without a badge), the total combinations were reduced to 20.

**Table 1:** Product attributes and attribute levels used in the conjoint analysis.

Attribute	Levels
Eco-labels/badge types (communicating disease resistance property)	No badge, sustainably grown, disease resistant, reduced agrichemicals, organic and climate resilient
Creative phrase	No phrase, friend of the environment (foe), and future of wine (fow)
Non-GMO logo	Present, absent

**Figure 3:**
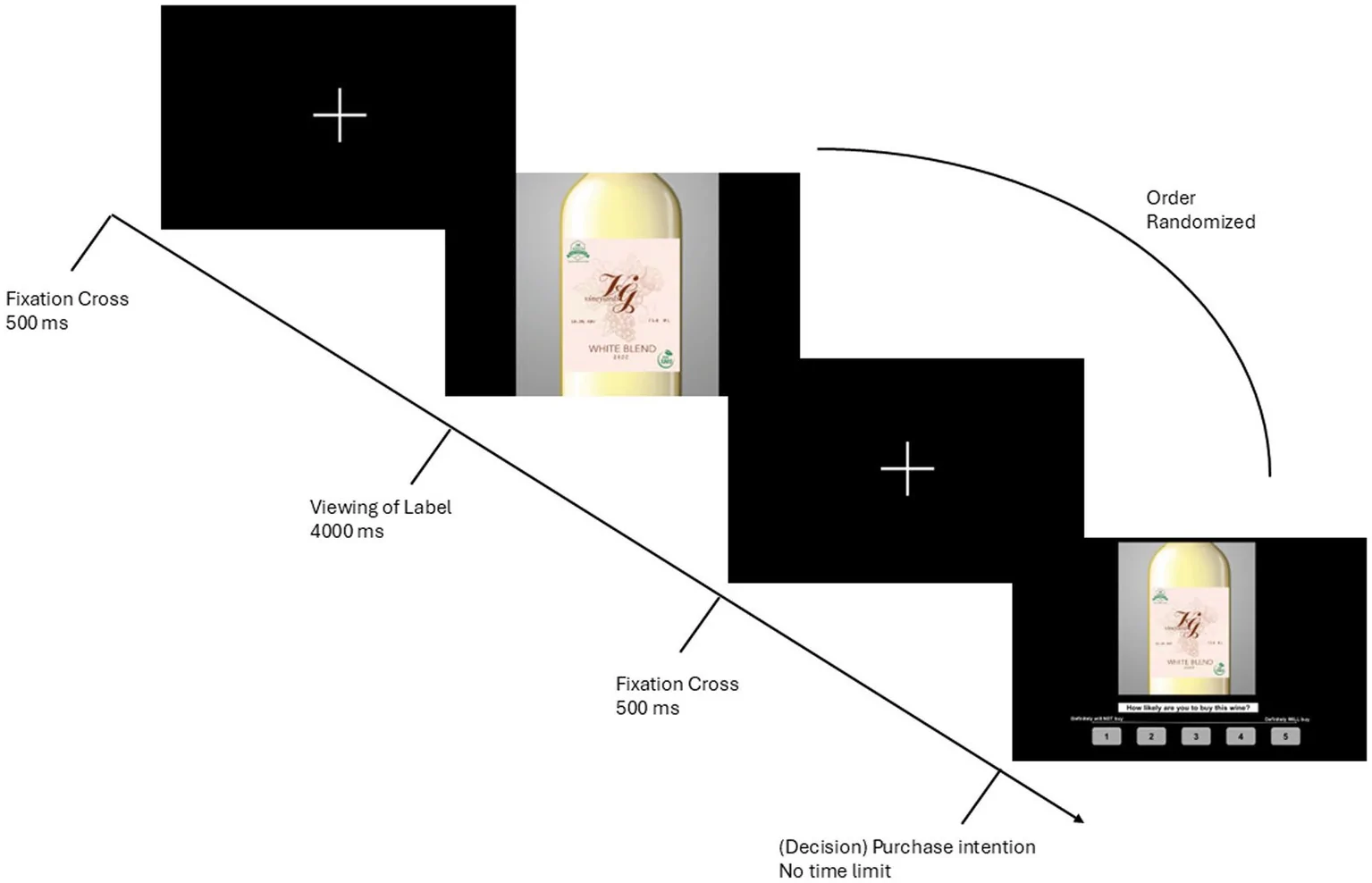
Illustration of the experimental trial structure.

To isolate the influence of message framing on consumer preferences, participants were explicitly told to assume all wine bottles were priced the same to eliminate price-based decision-making. Each participant was shown all 20 unique labels combinations randomly and asked to rate how likely they would purchase the wine based on the label on a five-point likert scale. To get reliable EEG data, each label was shown 20 times which resulted in a total of 400 trials per participant. To decrease fatigue and ensure participants were actively making decisions, they were encouraged to take a break halfway through the experiment. The two halves of the experiment were identical, and trials were randomized for each participant. Figure 3 illustrates the trial sequence, which began with a 500 ms fixation cross, followed by the label which was shown for 4,000 ms. Another fixation cross was presented for 500 ms and then participants were asked to rate their likelihood of purchase on a 5-point likert scale using the mouse. There was no time limit during the decision phase. The stimuli were presented on Tobii Pro lab where we simultaneously collected eye-tracking and EEG data while consumers viewed and made their decisions.

### Procedure

The process began by briefing the participants about the experiment and getting their written consent. Once all the equipment was set up, we began recording the data and requested participants to limit their movements to get clean data. The experiment began with clear instructions, emphasizing that while the experiment is hypothetical, participants should make choices as if they were making real purchasing decisions to reduce hypothetical bias. The introduction also contained a brief description of what disease-resistant crops were and their benefits. Participants were also briefed on the experiment set up, the attributes and their levels. A practice round was conducted to familiarize participants with the task before the formal experiment began. After the experiment, participants filled out an online questionnaire about their preferences for wine, knowledge of disease resistance and demographics variables.

Participants were seated approximately 68.5 cm from a 27-inch monitor (3,840 × 2,160 pixels at 60 Hz). The experiment room was sound-attenuated and free from visual distraction to improve the quality of data collected from the eye-tracker and EEG systems. We did not use a chinrest, but participants were seated comfortably and were instructed to minimize eye-blinks and body movements as much as possible during the experiment to reduce eye and muscle artifacts. The Tobii Pro Fusion screen-based eye-tracker was used to record participants’ eye movement while they viewed the stimuli with a sampling rate of 60 Hz. First, the participants’ eyes were calibrated to the eye-tracker using a standard 9-point calibration and validation procedure.

AntNeuro eego sport 64 channel gel-based cap was used to record EEG data. Electrodes were placed using the standard international 10–20 system and sampling rate was set at 500 Hz for high temporal resolution for event related potential (ERP) analysis. Impedances for all electrodes were kept below 20 kΩ before starting the recording for that session. EEG data was recorded continuously as participants viewed the wine labels and made their selections.

### Data pre-processing and analysis

The EEGLAB toolbox in MATLAB (The MathWorks, Inc., 2023) was used to pre-process and extract EEG data. The recorded data was first re-referenced to the average of the left and right mastoids. A 0.1–45 Hz bandpass filter was applied to remove low-frequency drift and high-frequency noise, and 60 Hz notch filter was applied to remove electrical line noise. Through manual inspection, noisy channels and flat channels were identified and interpolated using spherical spline interpolation. Continuous EEG data was then segmented into 2.5 s epochs, which included 500 ms pre-stimulus baseline and 2 s post-stimulus onset. Baseline correction was done using the 500 ms fixation cross period before stimulus onset. After epoching, messy trials with excessive channel noise or drifts were manually removed from the data.

Once epoched, we performed independent component analysis (ICA) on the data to remove eye-blinks, muscle artifacts and other horizontal eye movements. ERP components were then extracted from the data. We used the peak amplitudes from each ERP component for analysis. The time windows and electrode sites were selected based on the typical windows from previous literature.

P100 is a positive going ERP component, peaking at around 100 ms after the stimulus onset measured at the parietal and occipital regions (Herzberg et al., 2025; Zhu and Lv, 2023). In this study, we measured P100 between 0 and 200 ms at Oz, PO7, PO8, O1, and O2 electrode sites. P100 is an early signal that reflects visual processing activity and early attention which is often associated with unconscious processing of stimuli (Di Russo and Spinelli, 1999; Herzberg et al., 2025; Hillyard et al., 1998; Zhang et al., 2012).

N200 is a negative going ERP component that is related to unconscious processing of conflict, unfamiliarity or response inhibition (Byrne et al., 2022; Telpaz et al., 2015). This ERP component peaks between 200 and 350 ms after stimulus onset (Folstein and Van Petten, 2008). We measured N200 between 150 and 300 ms at Fz, FCz, C3, C4, FC1, FC2, F3, and F4.

The P300 ERP component is a positive going wave that typically peaks between 250 and 500 ms after the stimulus onset and is typically located in the posterior region of the brain (Polich, 2007). We measured P300 between 250 and 450 ms at Fz, C3, C4, Cz, P3, Pz, P4, PO7, Oz, and PO8. This ERP component reflects cognitive processing like working memory updates and allocation of attentional resources (Byrne et al., 2022; Wang and Han, 2014). Research has shown that stimuli that are salient, motivationally relevant or require increased evaluative processing generally result in higher P300 amplitudes (Nieuwenhuis et al., 2005).

Lastly, late positive potential (LPPs) is a late stage ERP component that generally reflect conscious emotional processing (Byrne et al., 2022) peaking between 500 and 800 ms and most prominent over the centro-parietal regions of the scalp (Hajcak et al., 2009; Hajcak and Foti, 2020). We measured LPPs between 600 and 2000 ms at C1, C2, CP1, CP2, P1, Pz, and P2 electrodes. It is related to sustained attentional and emotional processing of visual stimuli (Goto et al., 2019; Liu et al., 2012; Schupp et al., 2006).

**Figure 4:**
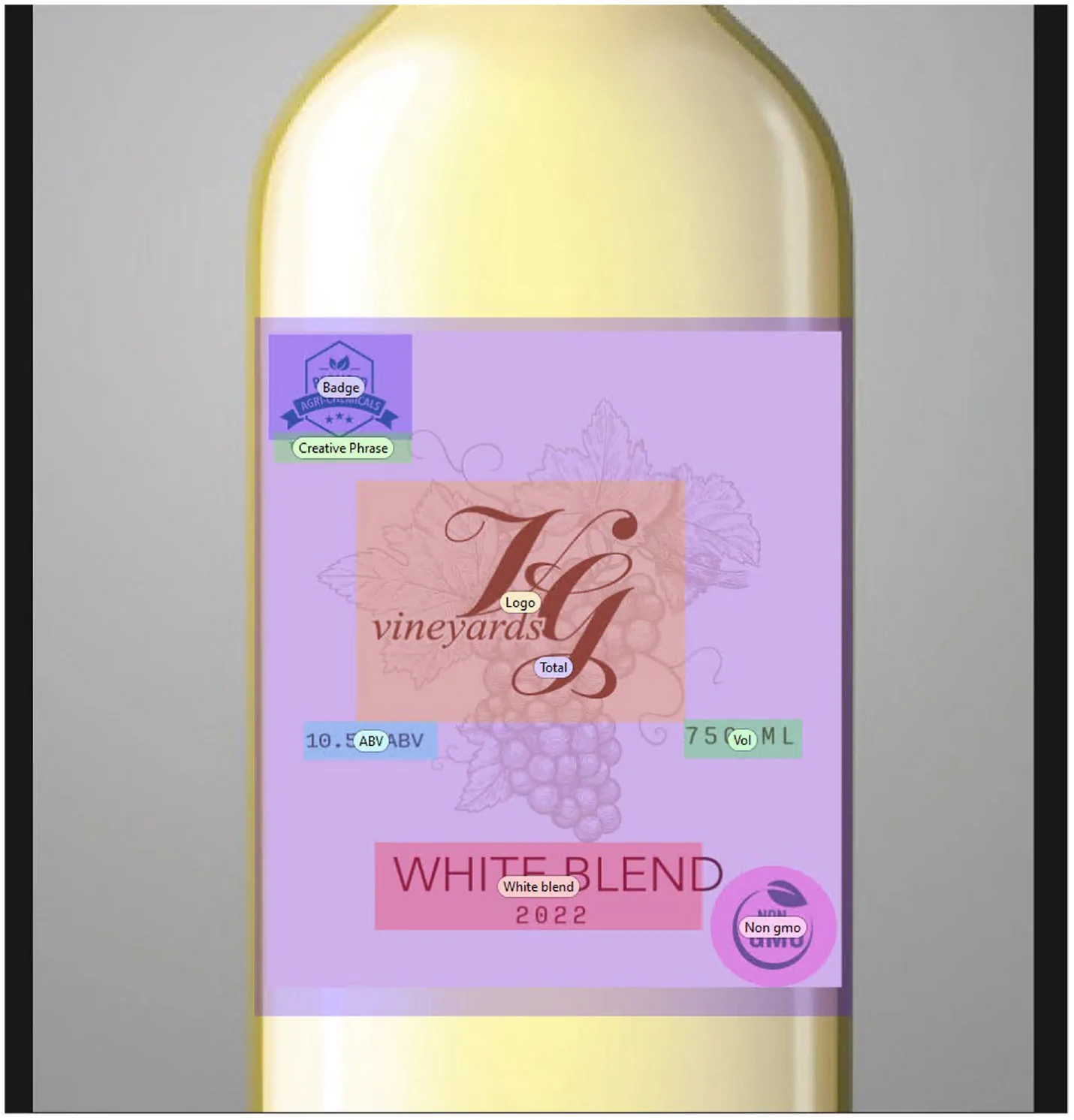
Eye-tracking areas of interest.

Tobii Pro lab was used to pre-process and extract eye-tracking data. The areas of interest (AOIs) were created after analyzing the heat maps of all participants’ data. Eight AOIs were identified: badge, creative phrase, brand, non-GMO logo, ABV, volume and total label (see Figure 4). For each AOI and trial we then extracted fixation duration (ms), fixation count, time to first fixation (ms) and duration of first fixation (ms) during the 4 s stimulus presentation time window. These measures were chosen based on previous eye-tracking studies (García-Madariaga et al., 2019; Krucien et al., 2017; Motoki et al., 2021; Van Loo et al., 2021; Zhu and Lv, 2023). To get the explicit responses, five AOIs were created on the decision screen that followed the stimulus presentation, one for each of the five Likert options. Mouse click data were extracted from the eye-tracking software to determine each participant’s trial level purchase intention.

The EEG, eye-tracking and behavioral response data was merged on Python, and the trial level data was aggregated by averaging over 20 trials per label. This gave us a data structure with 20 rows per participant which was subject to further data cleaning and analysis using Stata 17 (StataCorp, 2021).

Mixed-effects regression models were used to account for the repeated structure of the data, where each participant (i=1,…,N) rated multiple labels (j=1,…,J) . In order to account for within subject correlation and unobserved individual heterogeneity, parameters were estimated using a mixed-effects specification with random intercepts at the participant level, as in the general specification in Equation (1). The regressions with purchase intention (rating) as the dependent variable were estimated using mixed-effects Tobit regressions since the variable is continuous between 1 and 5 but is left censored at 1 and right censored at 5. Equations (2) and (5) are the estimating equations with purchase intentions as the dependent variable. The eye-tracking and EEG data were modeled using mixed-effects linear regressions, as in Equations (3) and (4).

The general model specification for observation j and individual i is:

$$y_{\mathrm{ij}}=X_{\mathrm{ij}}\beta +u_{i}+\varepsilon_{\mathrm{ij}}$$ (1)

where $X_{\mathrm{ij}}$ is a vector of label attributes, $u_{i}\sim N(0,\sigma_{u}^{2})$ captures individual specific effects and $\varepsilon_{\mathrm{ij}}\sim N(0,\sigma_{\varepsilon }^{2})$ is the idiosyncratic error term. All models were estimated by maximum likelihood estimation.

$$\begin{array}{l} \text{Ratin}g_{\mathrm{ij}}=\beta_{0}+\beta_{1}\text{Positivit}y_{\mathrm{ij}}+\beta_{2}\;\mathrm{FO}E_{\mathrm{ij}}+\beta 3_{3}\mathrm{FO}W_{\mathrm{ij}}\\ +\beta_{4}\;\text{nonGM}O_{\mathrm{ij}}+u_{i}+\varepsilon_{\mathrm{ij}} \end{array}$$ (2)

$$\mathrm{Eye}-\text{trackin}g_{\mathrm{ij}}=\alpha_{0}+\alpha_{1}\text{Positivit}y_{\mathrm{ij}}+u_{i}+\varepsilon_{\mathrm{ij}}$$ (3)

We used four eye-tracing variables: Total duration of fixations, number of fixations, time to first fixation and duration of first fixation. After estimating the coefficients, pairwise comparisons were run specifically to study differences in visual attention between high and low perceived positive badges.

$$\begin{array}{l} \mathrm{EE}G_{\mathrm{ij}}=\beta_{0}+\beta_{1}\text{Positivit}y_{\mathrm{ij}}+\beta_{2}\;\mathrm{FO}E_{\mathrm{ij}}+\beta 3_{3}\mathrm{FO}W_{\mathrm{ij}}\\ +\beta_{4}\;\text{nonGM}O_{\mathrm{ij}}+u_{i}+\varepsilon_{\mathrm{ij}} \end{array}$$ (4)

We used four ERP signals are the EEG variables: P100, N200, P300 and LPP.

$$\begin{array}{l} \text{Ratin}g_{\mathrm{ij}}=\beta_{0}+\beta_{1}\text{Positivit}y_{\mathrm{ij}}+\beta_{2}\;\mathrm{FO}E_{\mathrm{ij}}+\beta 3_{3}\mathrm{FO}W_{\mathrm{ij}}\\ +\beta_{4}\;\text{nonGM}O_{\mathrm{ij}}+\beta_{5}(\text{Positivit}y_{\mathrm{ij}}*\text{Eyetracking or}\;\mathrm{EEG}\;\text{metric}s_{\mathrm{ij}})\\ +u_{i}+\varepsilon_{\mathrm{ij}} \end{array}$$ (5)

## Results

### Post-hoc survey results for valence based classification

The mean perceived emotional valence ratings are presented in Table 2. The mean ratings for all the badges were above 4 (which is neutral on the 7-point scale), meaning none of the badges were perceived as negative overall. We conducted paired *t*-tests that revealed significant differences between the badges and helped in the categorization of the badges. Disease resistant was rated the lowest and it was significantly lower than all other badges. Reduced agrichemicals was rated significantly lower than sustainably grown, while climate resilient and organic were not rated statistically differently from each other. Based on these differences, the five badges were classified into three label categories of perceived positivity which is used in the analysis of the main experiment:

1. Neutral: no-badge
2. Low perceived positivity: disease resistant, reduced agrichemicals
3. High perceived positivity: sustainably grown, organic, climate resilient

**Table 2:** Mean rating of perceived emotional valence of the eco-labels (badges).

Label	Mean	SD
Sustainably grown	6.26	0.86
Organic	5.89	1.28
Climate resilient	5.48	1.19
Reduced agrichemicals	5.22	1.53
Disease resistant	4.56	1.63

Given that the badges used in the study were rated at or above neutral, we henceforth refer to perceived emotional valence as perceived positivity o refer to differences in the degree of positive emotional valence assigned to the labels.

### Summary statistics

The average age of the sample was between 50 and 59 years and 77% of the sample were female and the rest were male. The average annual household income was between $75,000 and $99,999 and mean education was a bachelor’s degree. The average household size was 2 people. The majority of participants (77%) were only slightly familiar with disease-resistant wine. The average purchase frequency was once a month and most of the sample (76.93%) stated that they had moderate knowledge regarding wine. Table 3 presents the summary statistics for the sample.

**Table 3:** Summary statistics of participants sociodemographic background information (*N* = 13).

Variable	Description	Mean	Standard deviation
Familiarity with disease resistance	Not familiar at all (1)	2.38	0.84
	Slightly familiar (2)		
	Moderately familiar (3)		
	Very familiar (4)		
	Extremely familiar (5)		
*Age*	21–29 (1)	3.69	1.49
	30–39 (2)		
	40–49 (3)		
	50–59 (4)		
	60–69 (5)		
	70 and older (6)		
*Gender*	Gender of participants, 1 = female, 0 = male	1.77	0.42
*Household income*	Under $25,000 (1)	4.23	1.48
	$25,000–$49,999 (2)		
	$50,000–$74,999 (3)		
	$75,000–$99,999 (4)		
	$100,000–$149,999 (5)		
	$150,000–$199,999 (6)		
	$200,000–$249,999 (7)		
	$250,000 and over (9)		
*Education*	Some high school or less (1)	6	1.52
	High school diploma or equivalent (2)		
	Some college, no degree (3)		
	Associate’s degree, occupational (4)		
	Associate’s degree, academic (5)		
	Undergraduate/bachelor’s degree (6)		
	Some graduate school (7)		
	Master’s degree (8)		
	Professional degree (9)		
	Doctoral degree (10)		
*Household people*	Number of people in the household of participants	2.15	1.03
*Race*	Race of the participant, 1 = white, 0 = otherwise	0.85	0.38
*Environment group*	Part of an environmental group, 1 = yes, 0 = no	0.08	0.28
*Wine purchase frequency*	Once a week (1)	3.15	1.1
	Once every other week (2)		
	Once a month (3)		
	Once every other month (4)		
	3–5 times a year (5)		
	1–2 times a year (6)		
*Wine expert*	No knowledge (1)	2.92	0.49
	Little knowledge (2)		
	Moderate knowledge (3)		
	Extensive knowledge (4)		
	Wine expert (5)		

### Conjoint analysis results

A mixed-effects regression was run to observe differences in reaction times based on perceived positivity (Table A1 and A2 in the Appendix). The regression revealed that the reaction times for labels with low-positivity badges were marginally higher than labels without a badge ( β = 0.099) and the reaction times between high positivity badges did not differ from the no badge (neutral) condition. The pairwise comparisons revealed that the reaction times for labels with low-positivity badges were significantly higher than labels with high positivity badges (difference = − 0.101).

Purchase intent was measured on a likert scale from 1 to 5 (least likely to buy to most likely to buy). Table 4 presents the mixed-effects Tobit regression results. Again, perceived positivity is a categorical variable with three levels: neutral (no badge), low (disease resistant and reduced agrichemicals badge) and high (sustainably grown, organic and climate resilient badges), constructed from the survey results. We found that badges with high perceived positivity had the strongest positive and significant impact on purchase intent (*β* = 0.80, *p* < 0.01) while badges with low perceived positivity did not significantly impact purchase intent compared to the base neutral perceived positivity (or no badge). Neither of the two creative phrases (“Friend of the Environment,” or “Future of Wine”) were significantly associated with purchase intent. Non-GMO is a binary variable, with 0 meaning it was not present and 1 denoting its presence on the label. The presence of the non-GMO logo on the label increased purchase intent at the 5% level of significance (*β* = 0.339).

**Table 4:** Mixed-effects Tobit regression on purchase intent.

Variable	Coeff
	(Std. Err)
Perceived positivity = low	−0.182
	(0.212)
Perceived positivity = high	0.800^***^
	(0.192)
Friend of the environment	0.116
	(0.131)
Future of wine	0.075
	(0.131)
Non-GMO	0.339^**^
	(0.138)
Constant	2.491^***^
	(0.210)
Random effects parameters	
Var(id)	0.174^**^
	(0.081)
Var(residuals)	0.665^***^
	(0.062)
Observations	260

Standard errors in parentheses.

^*^ *p* < 0.10, ^**^ *p* < 0.05, ^***^ *p* < 0.01.

### Eye-tracking results

We first estimated linear mixed-effects regressions for each eye-tracking measure (total duration of fixation, number of fixations, time to first fixation and duration of first fixation) on the badge AOI, regressed on the badge valence categories. Following this, we conducted pairwise post-estimation comparisons of the estimated marginal means across the different levels of perceived positivity. Table 5 reports the pairwise comparisons (Bonferroni-adjusted) between high and low perceived positivity on various eye-tracking metrics. We found that badges with high perceived positivity had significantly lower total duration of fixations and number of fixations compared to the badges with low perceived positivity. We did not find significant differences in the time to first fixation and duration of first fixation between the two types of badges, suggesting that they were noticed with equal speed. We mainly compared high and low perceived positivity, as the labels without a badge (i.e., neutral) would have lower duration and number of fixations because there was nothing for people to see. High positive badges resulted in lower total duration of fixations and number of fixations compared to low positive badges.

**Table 5:** Pairwise contrasts (Bonferroni-adjusted) of eye-tracking metrics by perceived positivity of the eco-labels (badges).

Measure	Difference perceived positivity (low–high)	Std. Err.
Total duration of fixations (s)	−0.115 ^***^	(0.036)
Number of fixations (#)	−0.396 ^***^	(0.115)
Time to first fixation (s)	0.032	(0.043)
Duration of first fixation (s)	−0.006	(0.012)

Results are pairwise contrasts of estimated marginal means from mixed-effects regressions with random intercepts for participants. Positive values indicate increases relative to comparison group (low perceived positivity). ^*^ *p* < 0.10, ^**^ *p* < 0.05, ^***^ *p* < 0.01.

**Figure 5:**
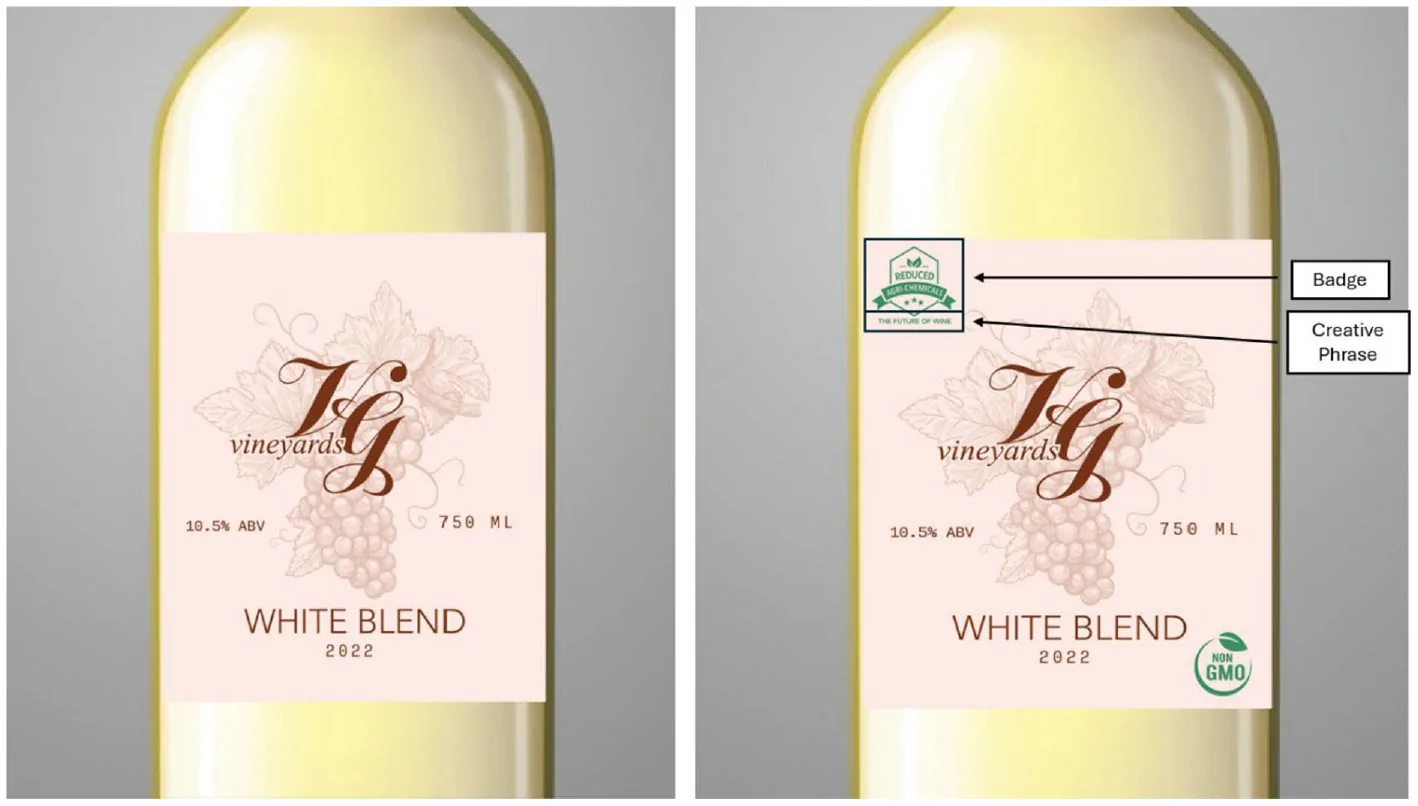
Heat maps depicting the total duration of fixations on the labels without a badge (left), with badge and no creative phrase (middle), and with a badge and creative phrase (right).

**Figure 6:**
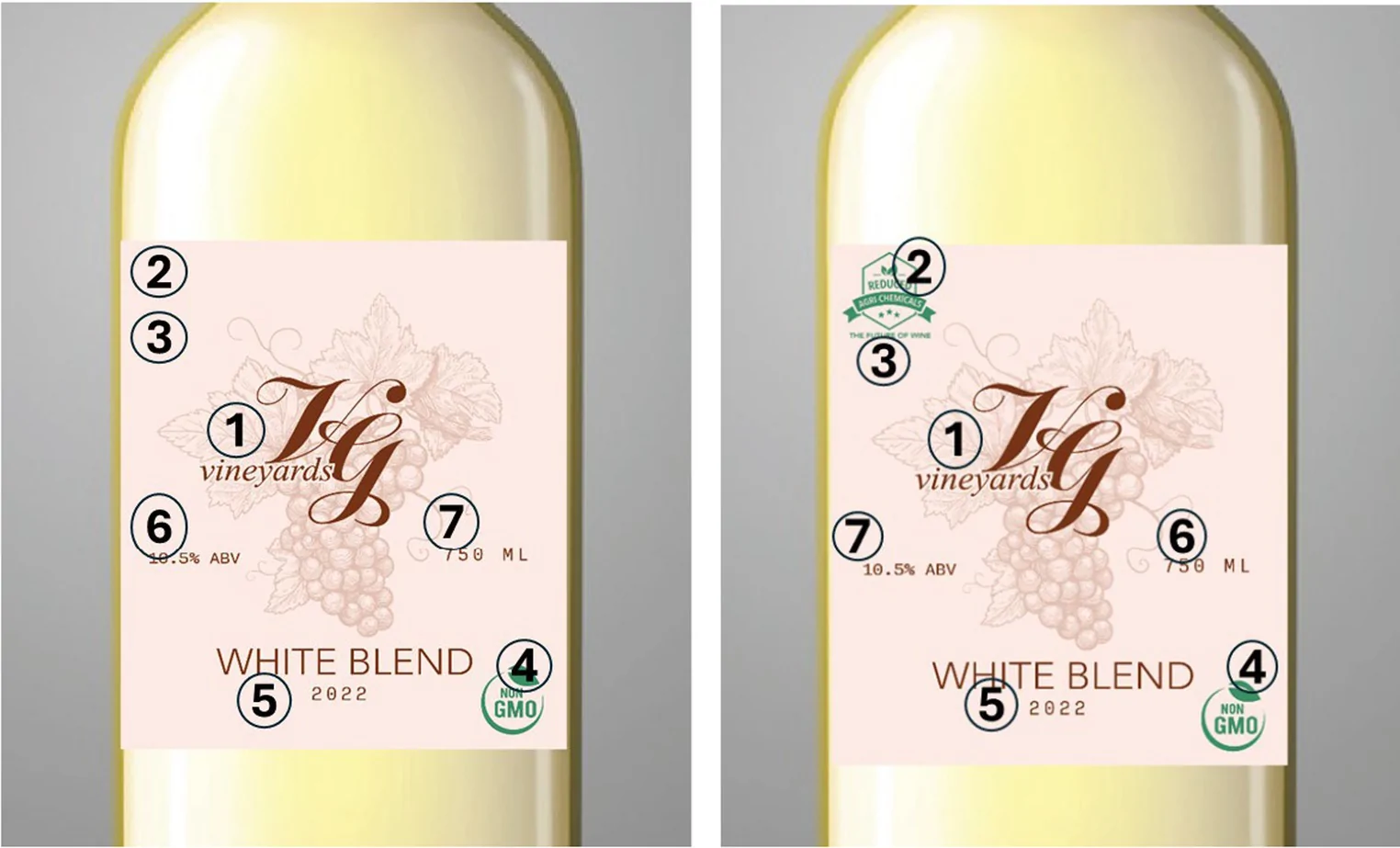
Gaze paths of participants for wine labels without a badge (left) and with low- and high-perceived positivity badges (right).

Figures 5, 6 present the heat maps for a subset of labels and gaze paths, respectively. The heat maps were based on the total duration of fixations and show the change on attention for labels without a badge and labels with a badge. When the labels did not have a badge (the leftmost label in Figure 5), most of the attention was towards the center brand. With a badge (the middle and rightmost label in Figure 5), fixation was most towards these badges. This suggested that the eco-labels were noticed, and they also helped focus attention on them. Figure 6 was constructed using the time to first fixation on the various AOIs. We noticed slight differences in the gaze patterns between labels with and without a badge, particularly the order is swapped for ABV and volume. We did not find differences in the gaze pattern between labels with high and low perceived positivity badges. Overall, we found that participants noticed the centre logo, the badge, creative phrase and then non-GMO in the bottom right, white blend, then the ABV or volume.

### EEG results

Mixed-effects linear regressions were estimated for each of the EEG ERP peak amplitudes -P100, N200, P300 and LPP within the selected time windows averaged across the electrodes selected. The results are presented in Table 6.

**Table 6:** Mixed-effects regression results on ERP peaks (P100, N200, P300 and LPP).

Variables	P100	N200	P300	LPP
Perceived positivity = low	0.446	0.318	3.479^***^	5.193^***^
	(0.421)	(0.562)	(0.483)	(0.733)
Perceived positivity = high	−0.081	−0.022	3.088^***^	4.261^***^
	(0.382)	(0.510)	(0.439)	(0.666)
Friend of the environment	0.134	0.667^*^	0.125	0.370
	(0.261)	(0.348)	(0.299)	(0.454)
Future of wine	−0.142	0.108	−0.145	−0.176
	(0.261)	(0.348)	(0.299)	(0.454)
Non-GMO	−0.351	−0.114	−0.491	−0.245
	(0.272)	(0.363)	(0.313)	(0.475)
Constant	14.570^***^	−11.296^***^	14.443^***^	20.082^***^
	(1.281)	(1.188)	(1.473)	(2.023)
Random effects parameters				
Var(id)	19.755^***^	15.564^***^	26.153^***^	48.427^***^
	(7.800)	(6.190)	(10.327)	(19.153)
Var(residuals)	2.650^***^	4.719^***^	3.493^***^	8.050^***^
	(0.238)	(0.425)	(0.314)	(0.724)
Observations	260	260	260	260

Standard errors in parentheses.

^*^ *p* < 0.10, ^**^ *p* < 0.05, ^***^ *p* < 0.01.

We did not find any significant effects of the label attributes on P100 ERP component. Moreover, we did not find any significant effects of low and high perceived positivity on P100 and N200 compared to the no-badge baseline. This may suggest that early visual response and conflict detection processes may not be strongly influenced by incremental changes in message valence. We found that only the ‘Friend of the Environment (foe)’ creative phrase increased N200 peaks at the 1% level of significance.

Both low and high perceived positivity badges elicited larger and significant P300 peaks compared to no badge. The magnitude increase was higher for badges with lower perceived positivity (*β* = 3.479) compared to the more positive badges (*β* = 3.088). This may suggest that regardless of the degree of positivity badges captured attention. Similarly, low and high positivity badges resulted in significantly higher LPPs compared to the baseline. Creative phrases and non-GMO logo did not significantly modulate P300 or LPP, suggesting that these attributes were less salient compared to the valence-based badges.

**Figure 7:**
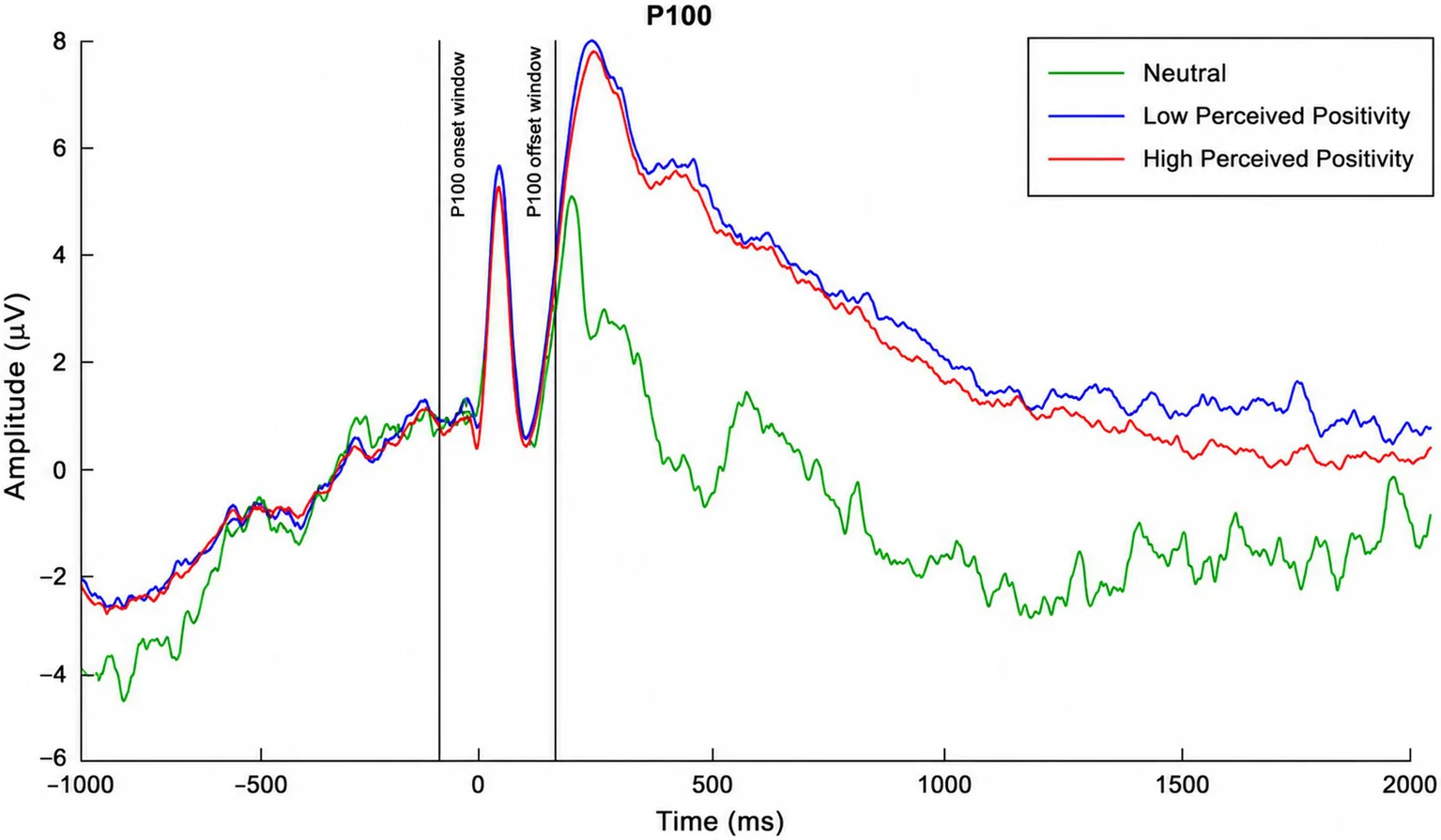
Grand average P100 waveforms (0–200 ms) across eco-labels (badges) with neutral, low, and high perceived positivity.

**Figure 8:**
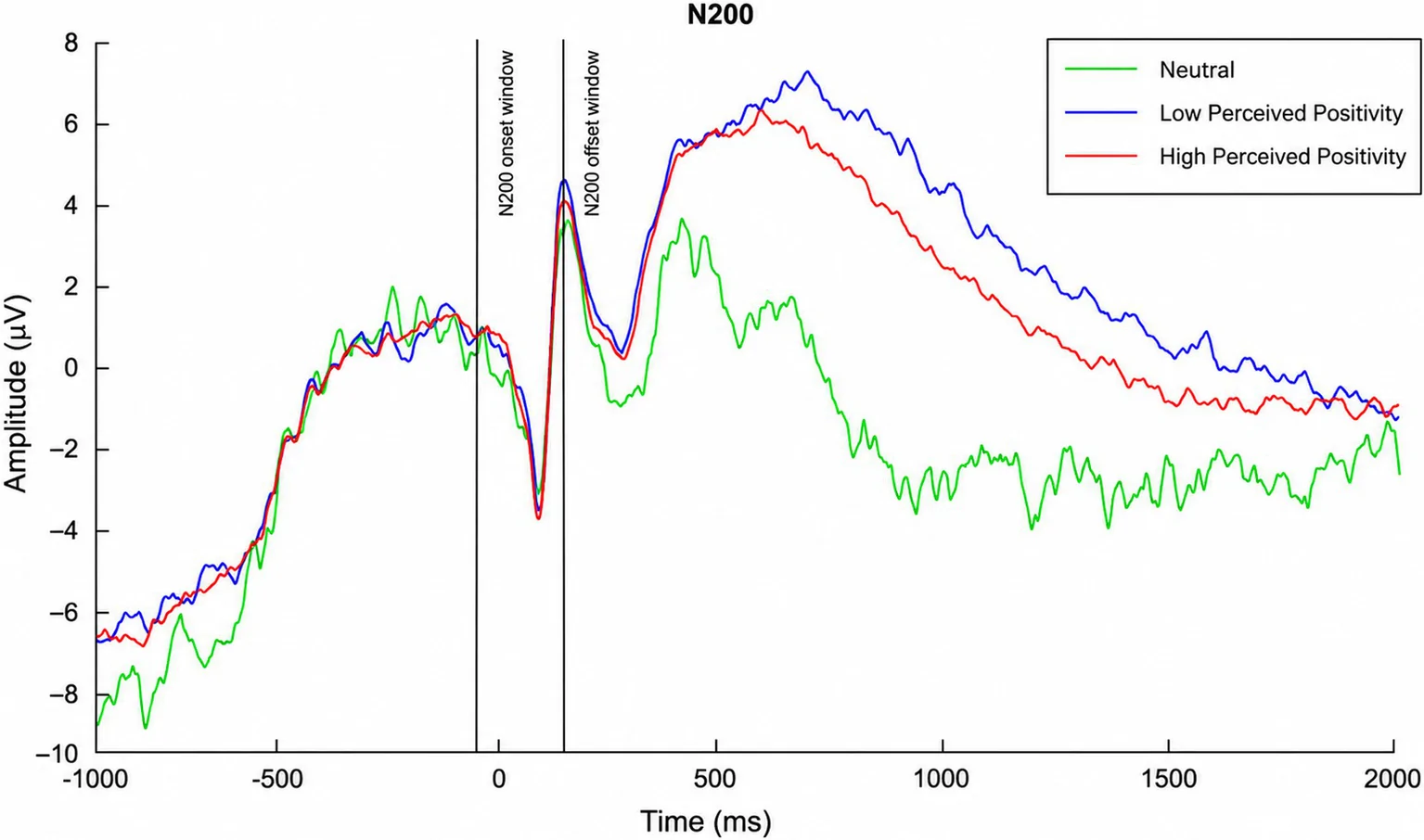
Grand average N200 waveforms (150–300 ms) across eco-labels (badges) with neutral, low and high perceived positivity.

**Figure 9:**
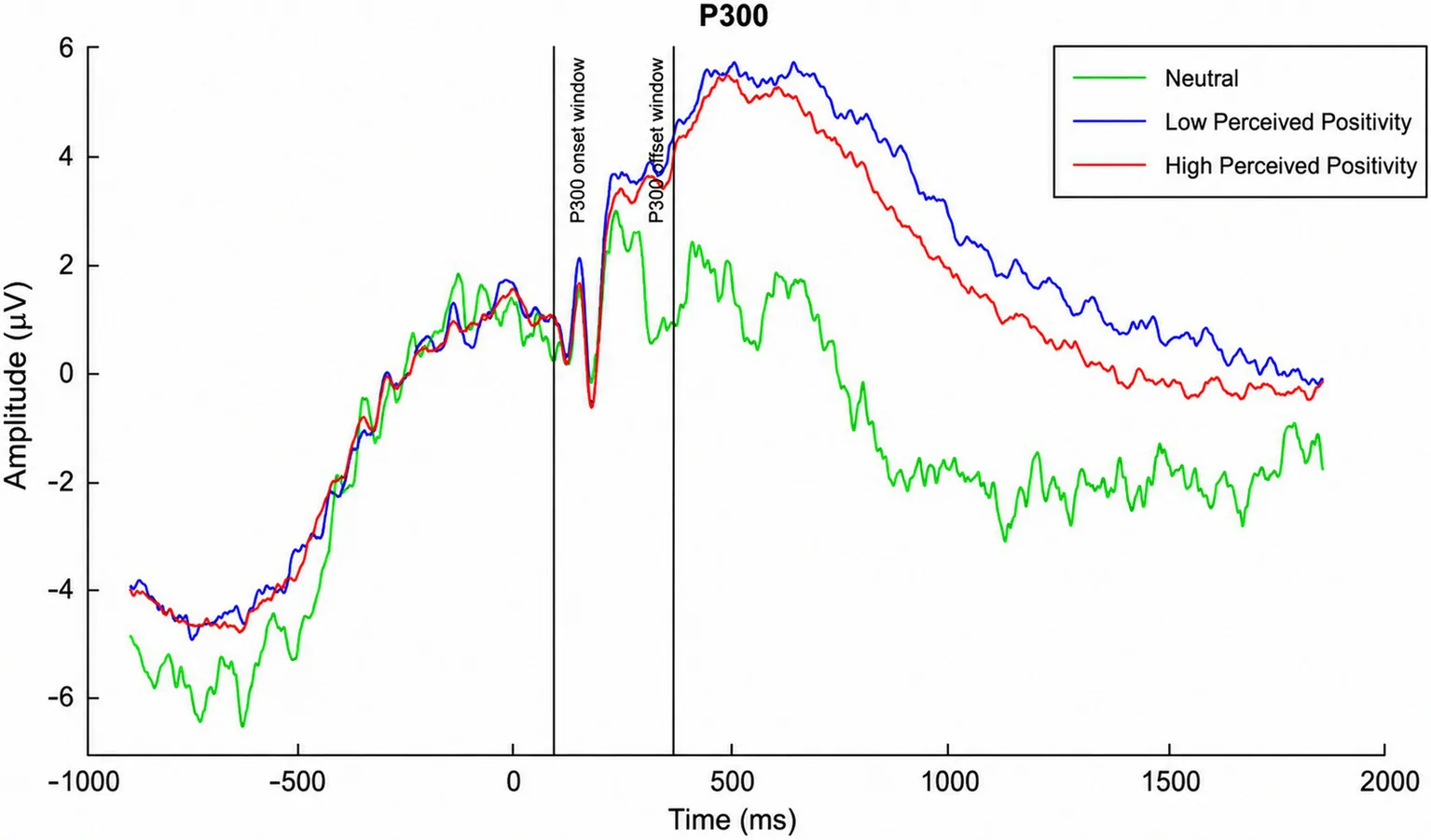
Grand average P300 waveforms (250–450 ms) across eco-labels (badges) with neutral, low and high perceived positivity.

**Figure 10:**
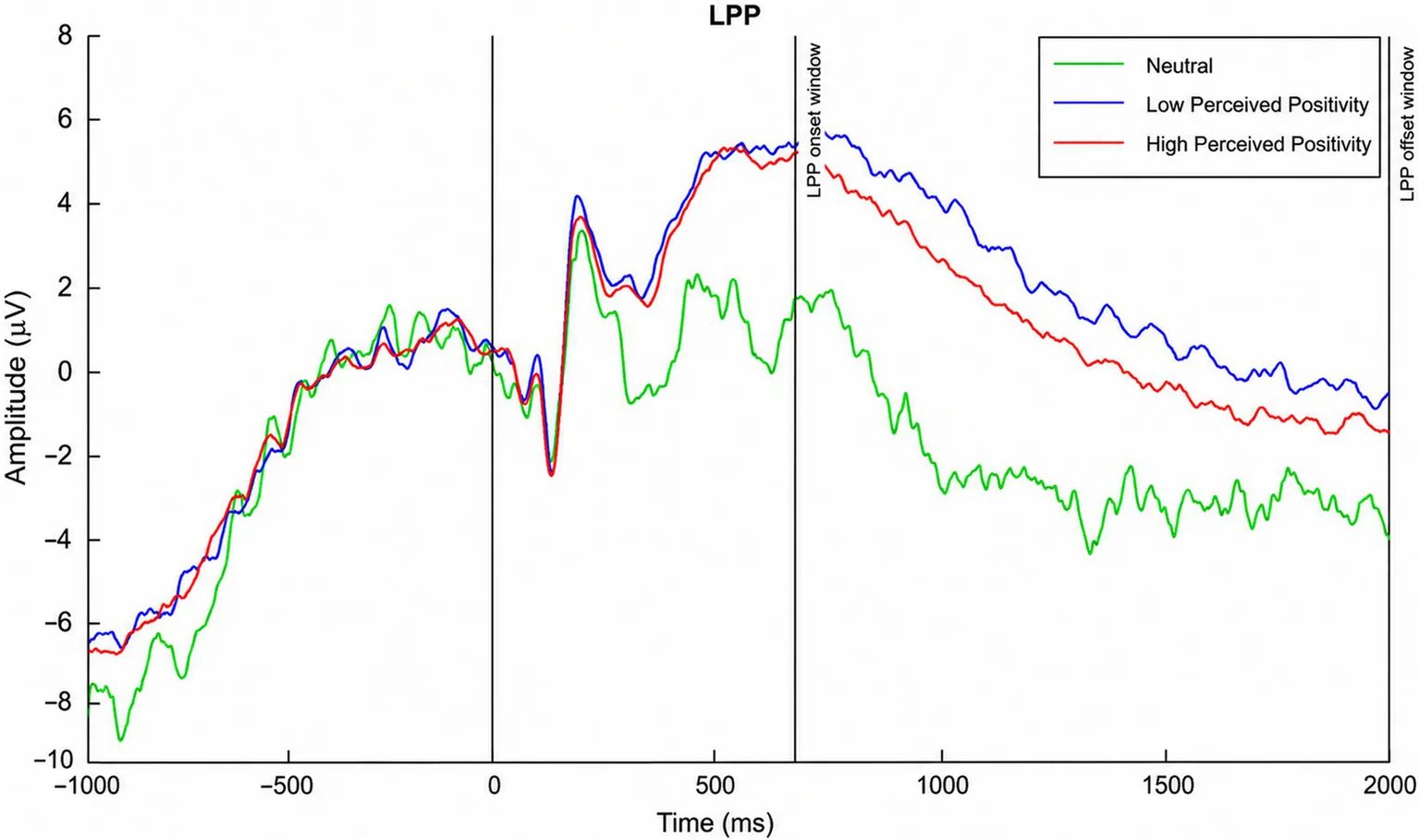
Grand average late positive potential (LPP) waveforms (600–2000 ms) across eco-labels (badges) with neutral, low and high perceived positivity.

**Figure 11:**
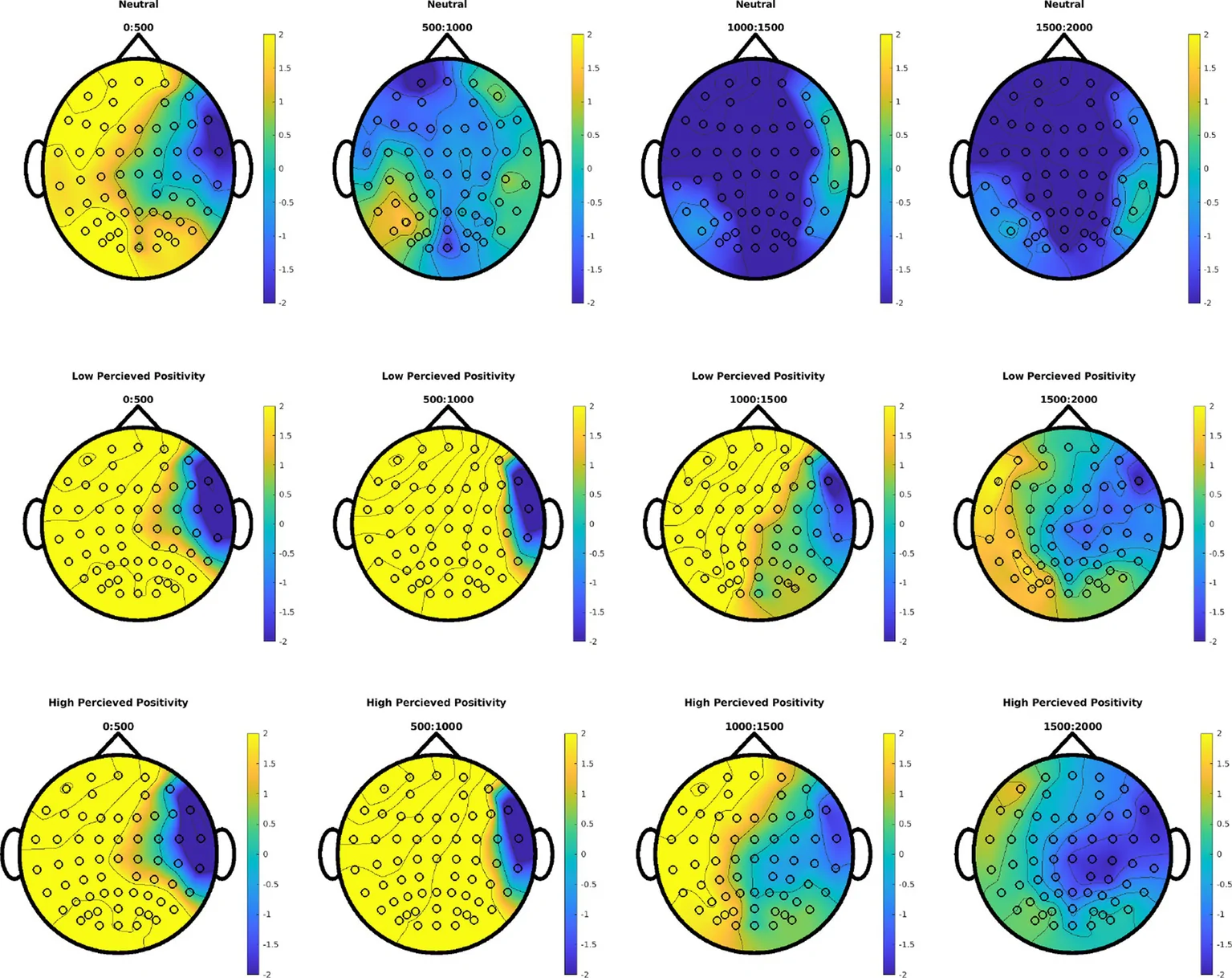
Scalp topographies in four 500 ms time windows between 0 and 2000 ms under the three badge perceived positivity categories – neutral (top), low perceived positivity (middle), and high perceived positivity (bottom).

Figures 7–10 illustrate the grand average ERP graphs for P100, N200, P300 and LPP, respectively for the three badge categories. We observed significant differences in the P300 and LPP amplitudes for badges with low and high perceived positivity compared to the no badge condition. Figure 11 is the scalp topography for neutral, low and high perceived positivity badge types across four successive 500 ms times windows (0–2000 ms). Across the three conditions we observed strong frontal and occipital activation between 0 and 500 ms, stronger for low and high positive badges compared to the no badge condition. The neutral (no) badge type showed little sustained activation indicated lower attention and engagement while the low and high positivity badges show strong sustained activation after 500 ms. The low perceived positivity badges showed stronger and more prolonged activation between 1500 and 2000 ms, suggesting more effortful emotional processing.

### Decision model results

We ran a mixed-effects Tobit regression using purchase intent as the dependent variable and the label attributes, significant eye-tracking metrics and ERP components as independent variables to help explain the purchase intent decision better. We estimated two models, one with only the main effects of the label attributes, relevant eye-tracking data and EEG measures. The other models included the interaction effects of badge valence with the same eye-tracking and EEG measures. These results are presented in Table 7.

**Table 7:** Mixed-effects Tobit regression results on purchase intent including label attributes, eye-tracking and EEG data.

Variables	Model 1	Model 2
Perceived positivity = low	−0.016	0.183
	(0.267)	(0.736)
Perceived positivity = high	0.941^***^	0.461
	(0.244)	(0.677)
Friend of the environment	0.180	0.182
	(0.132)	(0.130)
Future of wine	0.107	0.105
	(0.130)	(0.128)
Non-GMO	0.383^***^	0.377^***^
	(0.138)	(0.138)
Total duration of fixations (tdf)	−0.278^*^	-
	(0.160)	
Perceived positivity = neutral * tdf	-	−2.338
		(3.751)
Perceived positivity = low * tdf	-	−0.048
		(0.245)
Perceived positivity = high * tdf	-	−0.370^**^
		(0.172)
N200	−0.053^***^	-
	(0.016)	
Perceived positivity = neutral * N200	-	−0.046
		(0.041)
Perceived positivity = low * N200	-	−0.015
		(0.024)
Perceived positivity = high * N200	-	−0.079^***^
		(0.019)
P300	0.038^**^	-
	(0.015)	
Perceived positivity = neutral * P300	-	0.034
		(0.044)
Perceived positivity = low * P300	-	0.024
		(0.021)
Perceived positivity = high * P300	-	0.044^***^
		(0.016)
Constant	1.336^***^	1.529^**^
	(0.331)	(0.669)
Random effects parameters		
Var(id)	0.068^*^	0.065^*^
	(0.040)	(0.039)
Var(residuals)	0.652^***^	0.631^***^
	(0.061)	(0.059)
Observations	260	260

Standard errors in parentheses.

^*^ *p* < 0.10, ^**^ *p* < 0.05, ^***^ *p* < 0.01.

To avoid issues with multi-collinearity, a subset of eye-tracking and EEG metrics were selected. Number of fixations (nf) and total duration of fixations (tdf) on the badge AOI were highly correlated (*r* = 0.81) and only tdf significantly explained purchase intent (rating) at the 10% level of significance, nf was therefore dropped from the model. The ERP components also exhibited substantial inter-correlations, P300 and LPP peaks were strongly correlated (*r* = 0.88) and P300 and P100 were also correlated (*r* = 0.76). In addition, LPP did not significantly impact rating. When P100 and P300 both entered the model, neither was significance due to potential collinearity, however, when P300 was included alone it remained significant and had a greater explanatory power. Given these results, P300 was retained in the model, while N200 was included as an indicator of early conflict, as it was not significantly correlated with P300.

Model 1 showed similar results to the earlier conjoint analysis, we found that the presence of the non-GMO logo increased purchase intention significantly (*β* = 0.383). The creative phrases did not significantly affect purchase intent. Further, badges with high perceived positivity increased purchase intent significantly more than no badge (*β* = 0.941), while the low positivity badges were not significant. Lower total duration of fixations, lower N200 amplitudes and higher P300 amplitudes were associated with higher purchase intent.

Model 2 included the interactions between message valence, eye-tracking and EEG measures. Once the interactions were included, the direct effect of message valence became insignificant. Consistent with Model 1, the presence of non-GMO increased purchase intent while the creative phrases were not significant. We also found that increased duration of fixations on badges with high perceived positivity decreased their purchase intent.

Consistent with expectations, higher N200 peaks for low and high positivity badges decreased purchase intent. However, the effect was significantly different (from no badge) for high-positivity messages (*β* = −0.079). We also found that larger P300 amplitudes for high-positivity messages significantly increased purchase intent (*β* = 0.044), while the low positivity messages did not have a significant effect.

The difference in the results between the two models could suggest that the valence of the badges has less direct effects and is shaped by attention and cognitive evaluation which in turn influences purchase intent.

## Discussion

This study investigated the impact of perceived emotional valence of eco-labels for novel food products, particularly wines made from disease-resistant grapes on consumer preferences, visual attention, and neural processing. This research integrated self-reported purchase intent measures, eye-tracking and EEG to investigate the influence of perceived valence on the way consumers process sustainability-related information and how it shapes their purchase decisions.

Consistent with previous literature that has shown that positive environmental messages increase attitudes towards pro-social behavior and willingness to pay (Carfora et al., 2022; Lee and Cho, 2022; Weisstein et al., 2024), we found that badges with high perceived positivity (sustainably grown, organic and climate resilient) increased purchase intent significantly compared to low perceived positivity badges (disease resistant and reduced agrichemicals) which were not significantly associated with purchase intent. The preference toward more positive labels aligns with the sustainability liability effect (Turut, 2024) where unfamiliar or technically framed labels can appear less trustworthy or increase consumer skepticism.

The presence of non-GMO logo increased purchase intent, aligning with previous qualitative and focus group research on skepticism regarding the technology used to develop these grapes (Borrello et al., 2024). Although the ‘*Friend of the Environment*’ phrase was associated with a significant N200 response, the creative phrases were included as supplementary communication cues rather than the primary experimental manipulation. Moreover, neither creative phrase significantly influenced purchase intention in the behavioral or integrated models. Therefore, subsequent interpretation focuses on the perceived positivity of the eco-labels, which constituted the central research question of this study. This aligns with previous research suggesting that the effectiveness of multiple labels or eco-messages depends on their perceived coherence and complementarity of information. For example, previous research on multi-labeling has found that multi-labeling can backfire and decrease moral satisfaction when the labels are conflicting in their valence and orientation (Dorisse et al., 2025). Similarly, Pérez y Pérez et al. (2020) found that pairing two positive labels did not increase consumer valuation, especially when consumers perceived the labels to be substitutes. These results combined with the increase in N200 ERPs for the “friend of the environment” creative phrase could suggest a miss-match in expectations and rise in conflict, explaining the insignificant results from the conjoint analysis.

The eye-tracking and reaction time results revealed that badges with high perceived positivity attracted shorter fixation durations, fewer number of fixations and shorter reaction times. These results may seem counterintuitive at first glance but align with the processing fluency where stimuli which are easier to understand or more familiar require less cognitive effort and therefore less visual attention or scrutiny (Chrobot, 2014). In contrast, the longer fixations and number of fixations on the badges with low perceived positivity may indicate higher cognitive effort due to unfamiliarity, which is consistent with negativity bias perspectives (Rozin and Royzman, 2001; Soroka et al., 2019). Similar results have been observed in the online contexts where consumer fixated longer on information that was negative or ambiguous (Chen et al., 2022; Kohout et al., 2023), and in linguistic studies where shorter dwell times were observed for texts which were perceived as positively valenced (Usée et al., 2020).

The EEG results provided further insights into the cognitive and emotional mechanisms underlying the differences in behavioral patterns for the different badge types. Consistent with the functional interpretation of ERP components in previous literature (Polich, 2007; Hajcak and Foti, 2020), the change in amplitudes observed across early (P100 and N200) and late (P300 and LPP) ERP components suggested that label valence influenced both automatic and evaluative processing in different ways.

We did not observe significant differences in the peak amplitudes for P100 and N200 across the different badges compared to no badge, indicating that initial visual attention and cognitive conflict were not strongly modulated by the degree of perceived positive valence. These results align with some earlier evidence that very early components are less sensitive to subtle emotional or semantic difference (Hillyard et al., 1998; Di Russo and Spinelli, 1999). Jin et al. (2017) observed that negatively framed messages attracted more attentional resources which resulted in higher P200 amplitudes and higher cognitive conflict and decision difficulty with larger N200 compared to positive messages. The absence of a significant N200 difference across label valence conditions suggests that none of the eco-labels evoked strong expectancy violations or cognitive conflict, implying that the messages were processed as relatively familiar or credible cues. Further, based on the emotional valence ratings, none of the badges were rated to have a negative perceived valence, which may also explain the absence of significant N200 amplitude changes. This interpretation is consistent with Jin et al. (2018), who reported smaller N2 amplitudes for eco-labeled compared to non-labeled foods, indicating reduced decisional conflict for sustainability cues perceived as positive or trustworthy.

In contrast, we found that the badge valence significantly modulates conscious processing with changes observed on P300 and LPP ERP components. We found that the P300 and LPP amplitudes were significantly larger for both low and high perceived positivity labels compared to the no-badge conditions, with stronger effects for the low positivity badges. Research has shown that stimuli that are salient, motivationally relevant or require increased evaluative processing generally result in higher P300 amplitudes (Nieuwenhuis et al., 2005). Particularly, in the consumer neuroscience literature studies have related the P300 ERP component to the evaluation of product cues, product related information and brand preferences (Telpaz et al., 2015). These findings indicate the presence of any eco-label increased motivational salience and evaluation processing of the wine label (Byrne et al., 2022; Telpaz et al., 2015). The larger P300 amplitudes observed for less positive badges, specifically disease resistant and reduced agrichemicals, suggest that these badges required more cognitive evaluation and attentional resources. These results are in line with the eye-tracking results where we observed increased visual attention to these badges. Similar patterns have been reported in message-framing studies where negatively or ambiguously valenced cues evoke greater decision difficulty and cognitive conflict (Jin et al., 2017; Wei et al., 2024). This result also resonates with the notion that emotionally uncertain information may increase processing depth as individuals attempt to reconcile mixed associations (Carretié et al., 2004; Lu et al., 2017).

LPP reflects sustained attention and emotional engagement (Hajcak et al., 2009), and larger amplitudes typically indicate deeper emotional elaboration. Recent literature has reported the role of LPP in the cognitive process of evaluative categorization during the late stage of online purchase decisions (Chen et al., 2010; Jin et al., 2017; Wang et al., 2016). While there is some evidence of positive messages eliciting larger LPPs compared to negatively framed messages (Jin et al., 2017), more generally LPPs are affected by the valence strength of the emotional content, meaning LPP alone is unable to differentiate between positive and negative attitudes towards the brand or product (Byrne et al., 2022). We observed significantly higher LPPs for both low and high positive badges, with numerically stronger effects for low positive badges. This may indicate that low positive labels may result in increased evaluation and emotional ambivalence. These results are in line with findings by Wei et al. (2024), who found greater LPPs for negatively framed environmental messages which conveyed more perceived risk and emotional arousal. This interpretation is also consistent with prior findings that novel or ambiguous sustainability cues demand greater attentional resources (Grebitus et al., 2015) and that valence strength along a continuum modulates P300 amplitude (Onishi and Nakagawa, 2019).

Finally, the integrated models systematically revealed how label attributes, visual attention and neural processing shaped consumer’s purchase intentions. Consistent with the behavioral data only model, we found that non-GMO logo significantly increased purchase intent and the creative phrases did not influence purchase intent. Model 1, which only included the main effects of the label attributes, eye-tracking and EEG data, the high positivity badges significantly increased purchase intent compared to the baseline no badge condition. Lower total fixation duration, N200 and higher P300 increase purchase intent, suggesting that fluent, low-conflict, and motivationally engaging processing jointly enhance preference. These results align with previous neuromarketing evidence that has linked reduced cognitive conflict and increased P300 to increased purchase intent (Jin et al., 2017; Telpaz et al., 2015).

In Model 2, which included the interaction of the eye-tracking and EEG signals with perceived positivity, we found that the main effect of badge valence was no longer significant. This indicated that the influence of the badge valence operated through visual attention and neural mechanisms. Further, only the interactions on high perceived positivity badge are significantly associated with purchase intent. The negative interaction effect between fixation duration and high positivity badges indicated that increased attention towards these cues could increase scrutiny and reduce persuasion, which is consistent with prior work showing that overemphasis on sustainability claims can elicit skepticism (Dorisse et al., 2025). A similar effect is also seen between the interaction on N200 and high positivity badges, where increased N200 amplitude reduced purchase intent, indicative of conflict or expectancy violation (Folstein and Van Petten, 2008; Byrne et al., 2022). Further, larger P300 amplitudes only for high positivity badges increased purchase intent, reflecting increase motivational salience and evaluative engagement (Polich, 2007; Telpaz et al., 2015). Therefore, badges with high perceived positivity increase purchase intent when they are processed fluently and do not give rise to conflict.

Taken together, this research contributes to the literature on sustainability communication, especially for novel products, and consumer neuroscience by studying the effects of valence as a continuum rather than a binary distinction between positive and negative frames. While prior work using loss-gain frames have shown that valence shapes pro-environmental attitudes (Zubair et al., 2020; Wei et al., 2024), our results showed that even small variations in the tones of eco-labels can alter cognitive and affective processing. We found that sustainability cues shape choice through automatic (N200) and more reflective (P300 and LPP) pathways which reinforce dual processing theories of decision making (Kahneman, 2011) that states individual judgement and decision making is shaped by two cognitive systems – System 1(automatic and intuitive) and System 2 (logical and evaluative).

For marketers, these results highlight the importance of designing eco-labels that are credible, understandable and balance the expectations of their consumers. Specifically, for wines made from disease-resistant grapes, eco-labels that consist of more positive and familiar words – such as sustainably grown and organic, can increase purchase intent among consumers. However, we find that even labels with high perceived positivity can decrease purchase intent if they seem abstract and unfamiliar, which may increase skepticism and cognitive conflict. From a policy standpoint, the results highlight the value in standardizing and certifying eco-labels to reduce cognitive overload and perceived risk. Such an effort to harmonize sustainability communication, particularly for emerging categories like disease-resistant wines, could increase consumer trust and market transparency. Establishing a single well-recognized certification for disease-resistant or low-input wines could make it easy for consumers to identify these products in an increasingly crowded marketplace, while supporting producers who adopt sustainable viticulture practices.

This research is not without its limitations. The sample size is small, with only 13 valid participants. While it is not uncommon to have smaller sample sizes when conducting research with EEG (Alvino et al., 2021; Jin et al., 2017; Khushaba et al., 2013), a larger sample size would enable more detailed analysis on heterogeneity effects. This study should be viewed as an exploratory investigation of the cognitive and attentional processes associated with perceived emotional valence of eco-labels. Although the integration of behavioral, eye-tracking, and EEG measures provides converging evidence across multiple data sources, the relatively small EEG sample limits the precision and generalizability of the estimated effects, particularly for the interaction models. Consequently, the findings are best interpreted as generating hypotheses regarding the mechanisms through which eco-label valence influences consumer decision-making rather than providing definitive evidence of these mechanisms. The study focused on a specific product – disease-resistant wines, which may evoke unique emotions. Future research can expand on this research with a larger sample size to study how degree of valence influences cognitive and emotional mechanisms for different product types more generally. Further, the categorization of the badges into the three groups was based on previous literature and a *post hoc* survey with only 27 participants. Although this approach allowed for an empirically informed grouping aligned with participants’ affective evaluations, the limited sample size restricts the representativeness of the valence ratings. Consequently, the valence categories represent normative or aggregate consumer perceptions of the badge stimuli rather than participant-specific affective evaluations during the experiment. Although this approach allowed us to create an empirically informed classification of the stimuli while avoiding potential demand effects from collecting valence ratings immediately before or after the EEG task, it limits our ability to directly attribute individual neural and attentional responses to each participant’s own subjective perception of emotional valence. The present findings generate two testable hypotheses for future research:

*H1*: Eco-labels with higher perceived positive valence require less visual attention (shorter fixation durations and fewer fixations) than eco-labels with lower perceived positive valence.

*H2*: Higher perceived positive valence is associated with greater motivational salience (P300) and lower cognitive conflict (N200), resulting in higher purchase intentions.

Future research should test these hypotheses using larger and more diverse samples to evaluate the robustness, generalizability, and heterogeneity of the observed relationships.

## Data Availability

The raw data supporting the conclusions of this article will be made available by the authors, without undue reservation.

## References

[ref1] Alvino L. Constantinides E. van der Lubbe R. H. J. (2021). Consumer neuroscience: attentional preferences for wine labeling reflected in the posterior contralateral negativity. Front. Psychol. 12, 1–15. doi: 10.3389/fpsyg.2021.688713, 34721140 PMC8551361

[ref2] Anagnostou E. Tsiakis T. Zervas I. (2025). Highlighting wine labels: a systematic literature review of dominant informational parameters as communicative elements. Beverages 11:12. doi: 10.3390/beverages11010012

[ref3] Ariely D. Berns G. S. (2010). Neuromarketing: the hope and hype of neuroimaging in business. Nat. Rev. Neurosci. 11, 284–292. doi: 10.1038/nrn2795, 20197790 PMC2875927

[ref4] Babich N. (2017). Z-shaped pattern for reading web content. Medium. Available online at: https://uxplanet.org/z-shaped-pattern-for-reading-web-content-ce1135f92f1c (Accessed November 15, 2025).

[ref5] Bojko A. (2013). Eye Tracking the User Experience: A Practical Guide to Research. Rosenfeld Media. Brooklyn, NY: Rosenfeld Media.

[ref6] Borrello M. Cembalo L. Vecchio R. (2021). Consumers’ acceptance of fungus resistant grapes: future scenarios in sustainable winemaking. J. Clean. Prod. 307:127318. doi: 10.1016/j.jclepro.2021.127318

[ref7] Borrello M. Vecchio R. Barisan L. Franceschi D. Pomarici E. Galletto L. (2024). Is wine perception influenced by sustainability information? Insights from a consumer experiment with fungus resistant grape and organic wines. Food Res. Int. 190:114580. doi: 10.1016/j.foodres.2024.114580, 38945566

[ref8] Buscher G. Cutrell E. Morris M. R. (2009). What do you see when you’re surfing? Using eye tracking to predict salient regions of web pages Proceedings of the SIGCHI Conference on Human Factors in Computing Systems. New York, NY: Association for Computing Machinery (ACM). doi: 10.1145/1518701.1518705

[ref9] Byrne A. Bonfiglio E. Rigby C. Edelstyn N. (2022). A systematic review of the prediction of consumer preference using EEG measures and machine-learning in neuromarketing research. Brain Inform. 9:27. doi: 10.1186/s40708-022-00175-3, 36376735 PMC9663791

[ref10] Carfora V. Morandi M. Catellani P. (2022). The influence of message framing on consumers’ selection of local food. Foods 11:1268. doi: 10.3390/foods11091268, 35563989 PMC9105981

[ref9001] Carretié L. Hinojosa J. A. Martín-Loeches M. Mercado F. Tapia M. (2004). Modulation of cognitive processing by emotional valence studied through event-related potentials in humans. Neuroscience Letters, 356, 1–4. doi: 10.1016/j.neulet.2003.10.014 14746887

[ref11] Chen M. Ma Q. Li M. Dai S. Wang X. Shu L. (2010). The neural and psychological basis of herding in purchasing books online: an event-related potential study. Cyberpsychol. Behav. Soc. Netw. 13, 321–328. doi: 10.1089/cyber.2009.0142, 20557253

[ref12] Chen T. Samaranayake P. Cen X. Qi M. Lan Y.-C. (2022). The impact of online reviews on consumers’ purchasing decisions: evidence from an eye-tracking study. Front. Psychol. 13:865702. doi: 10.3389/fpsyg.2022.865702, 35756238 PMC9216200

[ref13] Chrobot N. (2014). The role of processing fluency in online consumer behavior: evaluating fluency by tracking eye movements Proceedings of the Symposium on Eye Tracking Research and Applications. (ETRA proceedings). New York, NY: Association for Computing Machinery (ACM). 387–388. Doi: 10.1145/2578153.2583037

[ref14] Cucchiara C. Kwon S. Ha S. (2015). Message framing and consumer responses to organic seafood labeling. Br. Food J. 117, 1547–1563. doi: 10.1108/BFJ-07-2014-0261

[ref15] de Boer J. (2003). Sustainability labelling schemes: the logic of their claims and their functions for stakeholders. Bus. Strateg. Environ. 12, 254–264. doi: 10.1002/bse.362

[ref16] Delmas M. A. Gergaud O. (2021). Sustainable practices and product quality: is there value in eco-label certification? The case of wine. Ecol. Econ. 183:106953. doi: 10.1016/j.ecolecon.2021.106953

[ref17] Di Russo F. Spinelli D. (1999). Electrophysiological evidence for an early attentional mechanism in visual processing in humans. Vis. Res. 39, 2975–2985. doi: 10.1016/S0042-6989(99)00031-0, 10664797

[ref18] Dorisse A. Charry K. Parguel B. (2025). When multi-labelling backfires: the influence of sustainability FOP labels valence and orientation. Appetite 213:108055. doi: 10.1016/j.appet.2025.108055, 40354982

[ref19] Duckworth J. J. Randle M. McGale L. S. Jones A. Doherty B. Halford J. C. G. . (2022). Do front-of-pack ‘green labels’ increase sustainable food choice and willingness-to-pay in U.K. consumers? J. Clean. Prod. 371:133466. doi: 10.1016/j.jclepro.2022.133466

[ref20] Duerrschmid K. Danner L. (2018). “Chapter 12—eye tracking in consumer research,” in Methods in Consumer Research, Volume 2, eds. Ares G. Varela P. (Duxford, UK: Woodhead Publishing), 279–318.

[ref21] Fantechi T. Contini C. Marinelli N. Moriondo M. Costafreda-Aumedes S. (2025). Sustainable wine – for whom? Consumer preferences for different environmental labels. Wine Econ. Policy 14, 17–29. doi: 10.36253/wep-17712

[ref22] Fernández-Serrano P. Tarancón P. Bonet L. Besada C. (2022). Consumers’ visual attention and choice of ‘sustainable irrigation’-labeled wine: logo vs. text. Agronomy 12:685. doi: 10.3390/agronomy12030685

[ref23] Fischer D. Reinermann J.-L. Mandujano G. G. DesRoches C. T. Diddi S. Vergragt P. J. (2021). Sustainable consumption communication: a review of an emerging field of research. J. Clean. Prod. 300:126880. doi: 10.1016/j.jclepro.2021.126880

[ref24] Folstein J. R. Van Petten C. (2008). Influence of cognitive control and mismatch on the N2 component of the ERP: a review. Psychophysiology 45, 152–170. doi: 10.1111/j.1469-8986.2007.00602.x, 17850238 PMC2365910

[ref25] García-Madariaga J. Blasco López M.-F. Burgos I. M. Virto N. R. (2019). Do isolated packaging variables influence consumers’ attention and preferences? Physiol. Behav. 200, 96–103. doi: 10.1016/j.physbeh.2018.04.030, 29702121

[ref26] Goto N. Lim X. L. Shee D. Hatano A. Khong K. W. Buratto L. G. . (2019). Can brain waves really tell if a product will be purchased? Inferring consumer preferences from single-item brain potentials. Front. Integr. Neurosci. 13:19. doi: 10.3389/fnint.2019.00019, 31316357 PMC6611214

[ref27] Grazzini L. Rodrigo P. Aiello G. Viglia G. (2018). Loss or gain? The role of message framing in hotel guests’ recycling behaviour. J. Sustain. Tour. 26, 1944–1966. doi: 10.1080/09669582.2018.1526294

[ref28] Grebitus C. Roosen J. Seitz C. C. (2015). Visual attention and choice: a behavioral economics perspective on food decisions. J. Agric. Food Ind. Organ. 13, 73–81. doi: 10.1515/jafio-2015-0017

[ref30] Hajcak G. Foti D. (2020). Significance?... Significance! Empirical, methodological, and theoretical connections between the late positive potential and P300 as neural responses to stimulus significance: an integrative review. Psychophysiology 57:e13570. doi: 10.1111/psyp.13570, 32243623

[ref29] Hajcak G. Dunning J. P. Foti D. (2009). Motivated and controlled attention to emotion: time-course of the late positive potential. Clin. Neurophysiol. 120, 505–510. doi: 10.1016/j.clinph.2008.11.028, 19157974

[ref31] Herzberg L. Schräder J. Jo H.-G. Habel U. Wagels L. (2025). Identifying P100 and N170 as electrophysiological markers for conscious and unconscious processing of emotional facial expressions. Front. Behav. Neurosci. 18:1464888. doi: 10.3389/fnbeh.2024.1464888, 39917258 PMC11798883

[ref32] Hillyard S. A. Vogel E. K. Luck S. J. (1998). Sensory gain control (amplification) as a mechanism of selective attention: electrophysiological and neuroimaging evidence. Philos. Trans. R. Soc. Lond. Ser. B Biol. Sci. 353, 1257–1270. doi: 10.1098/rstb.1998.0281, 9770220 PMC1692341

[ref33] Hoffmann A. Stork P. Madysa M. Borgianni Y. (2025). How attractive is sustainability in products: a systematic review about eye-tracking studies on sustainability labels. J. Environ. Psychol. 101:102519. doi: 10.1016/j.jenvp.2025.102519

[ref34] Hou Y. Guo P. Kannan D. Govindan K. (2023). Optimal eco-label choice strategy for environmentally responsible corporations considering government regulations. J. Clean. Prod. 418:138013. doi: 10.1016/j.jclepro.2023.138013

[ref35] Jin J. Dou X. Meng L. Yu H. (2018). Environmental-friendly eco-labeling matters: evidences from an ERPs study. Front. Hum. Neurosci. 12, 1–9. doi: 10.3389/fnhum.2018.00417, 30369874 PMC6194287

[ref36] Jin J. Zhang W. Chen M. (2017). How consumers are affected by product descriptions in online shopping: event-related potentials evidence of the attribute framing effect. Neurosci. Res. 125, 21–28. doi: 10.1016/j.neures.2017.07.006 28734975

[ref37] Kahneman D. (2011). Thinking, fast and slow. New York, NY: Farrar, Straus and Giroux, 499.

[ref38] Kahneman D. Tversky A. (1979). Prospect theory: an analysis of decision under risk. Econometrica 47, 263–291. doi: 10.2307/1914185

[ref39] Katz M. Campbell B. Liu Y. (2019). Local and organic preference: logo versus text. J. Agric. Appl. Econ. 51, 328–347. doi: 10.1017/aae.2019.4

[ref9002] Khushaba R. N. Wise C. Kodagoda S. Louviere J. Kahn B. E. Townsend C. (2013). Consumer neuroscience: Assessing the brain response to marketing stimuli using electroencephalogram (EEG) and eye tracking. Expert Systems with Applications, 40, 3803–3812. doi: 10.1016/j.eswa.2012.12.095

[ref40] Kohout S. Kruikemeier S. Bakker B. N. (2023). May I have your attention, please? An eye tracking study on emotional social media comments. Comput. Hum. Behav. 139:107495. doi: 10.1016/j.chb.2022.107495

[ref41] Krucien N. Ryan M. Hermens F. (2017). Visual attention in multi-attributes choices: what can eye-tracking tell us? J. Econ. Behav. Organ. 135, 251–267. doi: 10.1016/j.jebo.2017.01.018

[ref42] Lee J. Cho M. (2022). The (in)congruency effects of message framing and image valence on consumers’ responses to green advertising: focus on issue involvement as a moderator. J. Mark. Commun. 28, 617–636. doi: 10.1080/13527266.2021.1900893

[ref44] Liu C. Sharma C. Xu Q. Gonzalez Viejo C. Fuentes S. Torrico D. D. (2022). Influence of label design and country of origin information in wines on consumers’ visual, sensory, and emotional responses. Sensors 22:2158. doi: 10.3390/s22062158, 35336334 PMC8949006

[ref43] Liu Y. Huang H. McGinnis-Deweese M. Keil A. Ding M. (2012). Neural substrate of the late positive potential in emotional processing. J. Neurosci. 32, 14563–14572. doi: 10.1523/JNEUROSCI.3109-12.2012, 23077042 PMC3516184

[ref45] Liu Y. Zhao R. Xiong X. Ren X. (2023). A bibliometric analysis of consumer neuroscience towards sustainable consumption. Behav. Sci. 13, 298–298. doi: 10.3390/bs13040298, 37102812 PMC10136158

[ref46] Lockshin L. Corsi A. M. (2012). Consumer behaviour for wine 2.0: a review since 2003 and future directions. Wine Econ. Policy 1, 2–23. doi: 10.1016/j.wep.2012.11.003

[ref47] Loureiro M. L. Hine S. (2002). Discovering niche markets: a comparison of consumer willingness to pay for local (Colorado grown), organic, and GMO-free products. J. Agric. Appl. Econ. 34, 477–487. doi: 10.1017/S1074070800009251

[ref48] Luchs M. G. Naylor R. W. Irwin J. R. Raghunathan R. (2010). The sustainability liability: potential negative effects of ethicality on product preference. J. Mark. 74, 18–31. doi: 10.1509/jmkg.74.5.018

[ref49] Luck S. J. (2014). An Introduction to the Event-Related Potential Technique, Second Edition. Cambridge, MA: MIT Press.

[ref9005] Lu Y. Jaquess K. J. Hatfield B. D. Zhou C. Li H. (2017). Valence and arousal of emotional stimuli impact cognitive-motor performance in an oddball task. Biological Psychology, 125, 105–114. doi: 10.1016/j.biopsycho.2017.02.010, 28263878

[ref50] Mazzocchi C. Ruggeri G. Corsi S. (2019). Consumers’ preferences for biodiversity in vineyards: a choice experiment on wine. Wine Econ. Policy 8, 155–164. doi: 10.1016/j.wep.2019.09.002

[ref51] Motoki K. Saito T. Onuma T. (2021). Eye-tracking research on sensory and consumer science: a review, pitfalls and future directions. Food Res. Int. 145:110389. doi: 10.1016/j.foodres.2021.110389, 34112392

[ref52] Nieuwenhuis S. Aston-Jones G. Cohen J. D. (2005). Decision making, the P3, and the locus coeruleus—norepinephrine system. Psychol. Bull. 131, 510–532. doi: 10.1037/0033-2909.131.4.510 16060800

[ref53] Onishi A. Nakagawa S. (2019). How does the degree of valence influence affective auditory P300-based BCIs? Front. Neurosci. 13:45. doi: 10.3389/fnins.2019.00045, 30837822 PMC6390079

[ref54] Orquin J. L. Mueller Loose S. (2013). Attention and choice: a review on eye movements in decision making. Acta Psychol. 144, 190–206. doi: 10.1016/j.actpsy.2013.06.003 23845447

[ref55] Ozkara B. Y. Bagozzi R. (2021). The use of event related potentials brain methods in the study of conscious and unconscious consumer decision making processes. J. Retail. Consum. Serv. 58:102202. doi: 10.1016/j.jretconser.2020.102202

[ref56] Ozturk E. Kilic B. Cubero Dudinskaya E. Naspetti S. Solfanelli F. Zanoli R. (2023). Message in a bottle: an exploratory study on the role of wine-bottle design in capturing consumer attention. Beverages 9:Article 2. doi: 10.3390/beverages9020036, 30654563

[ref57] Pedneault K. Provost C. (2016). Fungus resistant grape varieties as a suitable alternative for organic wine production: benefits, limits, and challenges. Sci. Hortic. 208, 57–77. doi: 10.1016/j.scienta.2016.03.016

[ref58] Pérez y Pérez L. Gracia A. Barreiro-Hurlé J. (2020). Not seeing the forest for the trees: the impact of multiple labelling on consumer choices for olive oil. Foods 9:186. doi: 10.3390/foods9020186, 32069987 PMC7074561

[ref59] Polich J. (2007). Updating P300: an integrative theory of P3a and P3b. Clin. Neurophysiol. 118, 2128–2148. doi: 10.1016/j.clinph.2007.04.019, 17573239 PMC2715154

[ref60] Rahman I. Stumpf T. Reynolds D. (2014). A comparison of the influence of purchaser attitudes and product attributes on organic wine preferences. Cornell Hosp. Q. 55, 127–134. doi: 10.1177/1938965513496314

[ref61] Reutskaja E. Nagel R. Camerer C. F. Rangel A. (2011). Search dynamics in consumer choice under time pressure: an eye-tracking study. Am. Econ. Rev. 101, 900–926. doi: 10.1257/aer.101.2.900

[ref62] Richelli L. Arioli M. Canessa N. (2025). Neurosustainability: a scoping review on the neuro-cognitive bases of sustainable decision-making. Brain Sci. 15:678. doi: 10.3390/brainsci15070678, 40722271 PMC12293750

[ref63] Ropret Homar A. Knežević Cvelbar L. (2021). The effects of framing on environmental decisions: a systematic literature review. Ecol. Econ. 183:106950. doi: 10.1016/j.ecolecon.2021.106950

[ref64] Rozin P. Royzman E. B. (2001). Negativity bias, negativity dominance, and contagion. Personal. Soc. Psychol. Rev. 5, 296–320. doi: 10.1207/S15327957PSPR0504_2

[ref65] Russell J. A. (1980). A circumplex model of affect. J. Pers. Soc. Psychol. 39, 1161–1178. doi: 10.1037/h0077714

[ref66] Schupp H. T. Flaisch T. Stockburger J. Junghöfer M. (2006). Emotion and attention: event-related brain potential studies. Prog. Brain Res. 156, 31–51. doi: 10.1016/S0079-6123(06)56002-9, 17015073

[ref67] Seo Y. Nakao T. Nakada Y. (2025). An EEG study on visual imagery and consumer acceptance of upcycled food packaging. Retail Mark. Rev. 21, 69–86. doi: 10.5281/zenodo.17341374

[ref68] Sogari G. Mora C. Menozzi D. (2016). Factors driving sustainable choice: the case of wine. Br. Food J. 118, 632–646. doi: 10.1108/BFJ-04-2015-0131

[ref69] Soroka S. Fournier P. Nir L. (2019). Cross-national evidence of a negativity bias in psychophysiological reactions to news. Proc. Natl. Acad. Sci. 116, 18888–18892. doi: 10.1073/pnas.1908369116, 31481621 PMC6754543

[ref9003] StataCorp LLC. (2021). Stata Statistical Software: Release 17. College Station, TX: StataCorp LLC.

[ref9004] Steede G. M. Yue C. Gallardo R. K. Parasuram U. (2026). Communicating viticultural innovation: How wine labels influence enthusiasts’ views of disease resistance. Journal of Applied Communications, 110, 1–27. doi: 10.4148/jac.20844

[ref70] Tait P. Saunders C. Dalziel P. Rutherford P. Driver T. Guenther M. (2019). Estimating wine consumer preferences for sustainability attributes: a discrete choice experiment of Californian sauvignon blanc purchasers. J. Clean. Prod. 233, 412–420. doi: 10.1016/j.jclepro.2019.06.076

[ref71] Telpaz A. Webb R. Levy D. J. (2015). Using EEG to predict consumers’ future choices. J. Mark. Res. 52, 511–529. doi: 10.1509/jmr.13.0564

[ref72] Testa F. Iraldo F. Vaccari A. Ferrari E. (2015). Why eco-labels can be effective marketing tools: evidence from a study on Italian consumers. Bus. Strateg. Environ. 24, 252–265. doi: 10.1002/bse.1821

[ref73] Thaler R. H. Tversky A. Kahneman D. Schwartz A. (1997). The effect of myopia and loss aversion on risk taking: an experimental test*. Q. J. Econ. 112, 647–661. doi: 10.1162/003355397555226

[ref74] Thøgersen J. Nielsen K. S. (2016). A better carbon footprint label. J. Clean. Prod. 125, 86–94. doi: 10.1016/j.jclepro.2016.03.098

[ref75] Tiboni-Oschilewski O. Abarca M. Santa Rosa Pierre F. Rosi A. Biasini B. Menozzi D. . (2024). Strengths and weaknesses of food eco-labeling: a review. Front. Nutr. 11:1381135. doi: 10.3389/fnut.2024.1381135, 38600991 PMC11005915

[ref76] Turut O. (2024). The power of silent sustainability: communication strategies for new sustainable products. Clean. Responsible Consum. 14:100220. doi: 10.1016/j.clrc.2024.100220

[ref77] Tversky A. Kahneman D. (1992). Advances in prospect theory: cumulative representation of uncertainty. J. Risk Uncertain. 5, 297–323. doi: 10.1007/BF00122574

[ref78] Ugaglia A. A. Niklas B. Rinke W. Moscovici D. Gow J. Valenzuela L. . (2021). Consumer preferences for certified wines in France: a comparison of sustainable labels. Wine Econ. Policy 10, 75–86. doi: 10.36253/wep-10382

[ref79] Usée F. Jacobs A. M. Lüdtke J. (2020). From abstract symbols to emotional (in-)sights: an eye tracking study on the effects of emotional vignettes and pictures. Front. Psychol. 11:905. doi: 10.3389/fpsyg.2020.00905, 32528357 PMC7264705

[ref80] Van Loo E. Caputo V. Nayga R. Seo H.-S. Zhang B. Verbeke W. (2015). Sustainability labels on coffee: consumer preferences, willingness-to-pay and visual attention to attributes. Ecol. Econ. 118, 215–225. doi: 10.1016/j.ecolecon.2015.07.011

[ref81] Van Loo E. J. Caputo V. Nayga R. M. Verbeke W. (2014). Consumers’ valuation of sustainability labels on meat. Food Policy 49, 137–150. doi: 10.1016/j.foodpol.2014.07.002

[ref82] Van Loo E. J. Grebitus C. Verbeke W. (2021). Effects of nutrition and sustainability claims on attention and choice: an eye-tracking study in the context of a choice experiment using granola bar concepts. Food Qual. Prefer. 90:104100. doi: 10.1016/j.foodqual.2020.104100, 38826717

[ref84] Wang G. Li J. Zhu C. Wang S. Jiang S. (2021). How do reference points influence the representation of the N200 for consumer preference? Front. Psychol. 12:645775. doi: 10.3389/fpsyg.2021.645775, 34248744 PMC8266263

[ref83] Wang J. Han W. (2014). The impact of perceived quality on online buying decisions: an event-related potentials perspective. Neuroreport 25, 1091–1098. doi: 10.1097/WNR.0000000000000233, 25037004

[ref85] Wang Q. Meng L. Liu M. Wang Q. Ma Q. (2016). How do social-based cues influence consumers’ online purchase decisions? An event-related potential study. Electron. Commer. Res. 16, 1–26. doi: 10.1007/s10660-015-9209-0

[ref86] Wei Q. Bao A. Lv D. Liu S. Chen S. Chi Y. . (2024). The influence of message frame and product type on green consumer purchase decisions: an ERPs study. Sci. Rep. 14:23232. doi: 10.1038/s41598-024-75056-2, 39369070 PMC11457498

[ref87] Weisstein F. L. Meyer J. Kershaw J. (2024). A matter of alignment? Effects of product types and environmental claim framing on consumer evaluation of sustainable foods. Bus. Strateg. Environ. 33, 1661–1674. doi: 10.1002/bse.3565

[ref9006] Zhang Q. Kong L. Jiang Y. (2012). The interaction of arousal and valence in affective priming: Behavioral and electrophysiological evidence. Brain Res. 1474, 60–72. doi: 10.1016/j.brainres.2012.07.023, 22820299 PMC3694405

[ref88] Zhu L. Lv J. (2023). Review of studies on user research based on EEG and eye tracking. Appl. Sci. 13:6502. doi: 10.3390/app13116502

[ref89] Zubair M. Wang X. Iqbal S. Awais M. Wang R. (2020). Attentional and emotional brain response to message framing in context of green marketing. Heliyon 6:e04912. doi: 10.1016/j.heliyon.2020.e04912, 33005782 PMC7519354

