# Supplementary material for: Understanding the impact of perceived emotional valence of eco-labels for novel food products on consumer preferences, visual attention, and cognition – a multimodal study using EEG and eye tracking
Source: Front Psychol. 2026 Sep 8;17:1836306. doi: 10.3389/fpsyg.2026.1836306 (PMC13593434; doi:10.3389/fpsyg.2026.1836306)
Supplement: Supplementary file 1 [file Supplementary_File_1.docx]

# Appendix

**Table A1. Mixed-effects regression on reaction time**

|  | Coeff |
| --- | --- |
|  | (Std. Err) |
|  |  |
| Perceived positivity = low | 0.099^*^ |
|  | (0.053) |
|  |  |
| Perceived positivity = high | -0.003 |
|  | (0.050) |
|  |  |
| Constant | 1.078^***^ |
|  | (0.081) |
| Random effects parameters |  |
| Var(id) | 0.057^***^ |
|  | (0.026) |
|  |  |
| Var(residuals) | -0.055^***^ |
|  | (0.005) |
|  |  |
| Observations | 260 |

Standard errors in parentheses

^*^ *p* < 0.10, ^**^ *p* < 0.05, ^***^ *p* < 0.01

**Table A2. Pairwise comparisons of reaction time (mixed-effects model) between the three levels of perceived positivity of the eco-labels (badges)**

| **Comparison (Perceived Positivity)** | **Coefficient** | **Std. Error** | **95% Confidence Interval** |
| --- | --- | --- | --- |
| Low vs. Neutral (1 vs 0) | 0.099^*^ | 0.053 | [-0.006, 0.203] |
| High vs. Neutral (2 vs 0) | -0.003 | 0.050 | [-0.100, 0.095] |
| High vs. Low (2 vs 1) | -0.101^**^ | 0.033 | [-0.165, -0.037] |

**Table A3. Mixed-effects Tobit regression using continuous perceived positivity (Behavioral model)**

| Variable | Coeff. |
| --- | --- |
|  | (Std. Err.) |
| Eco-label perceived positivity (continuous) | 0.301^***^ |
|  | (0.033) |
| Friend of the Environment | 0.008 |
|  | (0.125) |
| Future of Wine | -0.032 |
|  | (0.125) |
| Non-GMO | 0.191 |
|  | (0.128) |
| Constant | 2.242^***^ |
|  | (0.190) |
| **Random effects parameters** |  |
| Var(id) | 0.175^**^ |
|  | (0.082) |
| Var(residuals) | 0.653^***^ |
|  | (0.061) |
| **Observations** | **260** |

Standard errors in parentheses

^*^ *p* < 0.10, ^**^ *p* < 0.05, ^***^ *p* < 0.01

**Table A4. Mixed-effects Tobit regression using continuous perceived positivity including eye-tracking and EEG measures (Integrated model)**

| Variable | Coeff. |
| --- | --- |
|  | (Std. Err.) |
| Eco-label perceived positivity (continuous) | 0.326^***^ |
|  | (0.033) |
| Friend of the Environment | 0.146 |
|  | (0.128) |
| Future of Wine | 0.063 |
|  | (0.125) |
| Non-GMO | 0.320^**^ |
|  | (0.131) |
| Total duration of fixations (TDF) | -0.431^***^ |
|  | (0.130) |
| N200 peak amplitude | -0.059^***^ |
|  | (0.016) |
| P300 peak amplitude | 0.034^**^ |
|  | (0.014) |
| Constant | 1.188^***^ |
|  | (0.322) |
| **Random effects parameters** |  |
| Var(id) | 0.067^*^ |
|  | (0.039) |
| Var(residuals) | 0.621^***^ |
|  | (0.058) |
| **Observations** | **260** |

Standard errors in parentheses

^*^ *p* < 0.10, ^**^ *p* < 0.05, ^***^ *p* < 0.01
